# Development and 5-year Evaluation of Diagnosis-Specific Protocols for Visual Neuro-Rehabilitation in a Multicenter Inpatient Rehabilitation Network

**DOI:** 10.1016/j.arrct.2022.100246

**Published:** 2022-11-17

**Authors:** Kevin E. Houston, Matthew Keilty, Caroline Collins, Ritika Trehan, Talia Mouldovan, Kim Stuckart, Nancy Engelhardt, Melanie Nadeau, Craig A. Rovito, Lotfi B. Merabet

**Affiliations:** a Spaulding Rehabilitation Hospital Cape Cod, Sandwich, MA; b Spaulding Hospital for Continuing Medical Care Cambridge, Cambridge, MA; c Spaulding Rehabilitation Hospital, Boston, MA; d Massachusetts Eye and Ear, Optometry and Vision Rehabilitation Service, Boston, MA; e Harvard Medical School, Department of Ophthalmology, Boston, MA; f Schepens Eye Research Institute, Boston, MA; g Department of Physical Medicine & Rehabilitation, Harvard Medical School, Boston, MA

**Keywords:** Cranial nerve diseases, Hemianopsia, Hemispatial neglect, Neurorehabilitation, Palsy, Rehabilitation, Saccadic eye movement

## Abstract

**Objective:**

To provide a retrospective evaluation of a new eye and vision rehabilitation care pathway in a U.S. multi-site inpatient rehabilitation network involving the occupational therapy (OT) staff and a consulting doctor of optometry (OD) specializing in vision rehabilitation.

**Design:**

Retrospective study.

**Setting:**

Two Inpatient Rehabilitation Facilities (IRFs) and 1 Long Term Acute Care Hospital (LTACH).

**Participants:**

There were 2083 records reviewed (44% women, avg. age 59 years). The most common diagnoses were hemispatial neglect (19.2%), homonymous field defects (18.5%), and oculomotor cranial nerve palsies (16.7%) (N=2083).

**Interventions:**

Clinical care was reviewed where diagnosis-specific protocols were developed and training was provided to OTs in order to reinforce OD-prescribed interventions during daily treatment sessions, including (1) third, fourth, and sixth ocular cranial nerve palsies (OCNPs) with prisms fitted for full time, postural adaptation training, and oculomotor re-education using pursuits, saccades, head-rotations, and binocular vision exercises including alternate cover and vergence; (2) homonymous hemianopia with training awareness of field loss, eccentric viewing, and fitting of Peli lens for optical field expansion; and (3) prism adaptation therapy (PAT) for left hemispatial neglect.

**Main Outcome Measures:**

Frequency of diagnoses.

**Hypothesis:**

Diagnoses with developed protocols were most common. Secondarily, feasibility and efficacy by anonymous OT survey.

**Results:**

2083 vision consults were performed over 5 years. The most common diagnoses were hemispatial neglect (n=399, 19.2%), homonymous field defects (n=386, 18.5%), and OCNPs (n=347, 16.7%). None of the OTs reported the protocols were infeasible and 63% (IQR 38%-69%) reported their patients benefited from the interventions. The survey suggested prism for OCNPs helped in 42%, and Peli lens and PAT both helped in 38%.

**Conclusions:**

Data support the feasibility of this inpatient eye and vision rehabilitation care pathway which may be used as a foundation for creating or refining similar programs elsewhere. Uniform administration of IRF-based visual neuro-rehabilitation care could provide a substrate for future clinical trials to evaluate efficacy.

Inpatient rehabilitation facilities (IRFs) in the United States provide 24-hour medical supervision and intensive rehabilitation therapy services for patients who have sustained a major injury or illness and cannot immediately transition home. IRFs are staffed and directed by physicians (typically physiatrists) with integrated neuropsychology, occupational, physical, speech therapy, nursing, social work, and case management services. There are approximately 1100 IRFs in the U.S. with an average length of stay of approximately 2 weeks.^1^

The prevalence of vision problems in U.S. IRFs has not been investigated, but it is likely substantial given the high proportion of the patients admitted after stroke or other brain injuries. Estimates from similar facilities in the United Kingdom (U.K.) and the U.S. Veterans Administration System suggest 60%-70% of inpatients may have visual problems,^2^, ^3^, ^4^, ^5^, ^6^, ^7^ strabismus,^2^^,^^6^^,^^8^^,^^9^ hemianopia,^9^ and hemispatial neglect.^10^

Vision rehabilitation includes, but is not limited to, the use of education, compensatory strategies, exercises, task modification, assistive technology, and visual aids to improve function and quality of life issues attributable to the visual loss.^11^ In visual neuro-rehabilitation, Fresnel press-on prisms are an inexpensive visual aid often prescribed for strabismus, and sometimes for homonymous field defects to expand the hemianopic field. Fresnel press-on prisms can be custom-fitted to the patient's own frame or a plano frame at the time of examination with only a pair of scissors and can be easily adjusted as the patient recovers. Press-on prisms have been shown to be well accepted/tolerated during the initial fitting in 94% of inpatients with strabismus and 74% with hemianopia.^12^ Reports on long-term acceptance/tolerance in outpatient clinics has been reported at 80%^13^ for strabismus, and about 40% for hemianopia.^14^ The Peli prism design for hemianopia is supported by a double-blind multicenter randomized controlled trial (RCT) which found better mobility questionnaire scores compared with sham treatment.^14^ In addition to prisms, training may be provided to help compensate for the impairment including adaptive positioning of the head and/or adjusting reading material and gaze direction to reduce double vision or the effect of the field loss.^15^^,^^16^ Oculomotor therapies (sometimes called orthoptic therapies) which aim to restore impaired eye motility via repetitive eye movements into the paretic motor field, or improving vergence eye movements, are frequently employed despite limited evidence regarding their efficacy for ocular cranial nerve palsies (OCNPs).

Visual neuro-rehabilitation is a subspecialty in this field and may involve doctors of optometry (ODs) who are residency-trained in low vision and/or binocular vision therapy, occupational therapists (OT) with advanced training in vision impairments, orthoptists, and certified low vision therapists. In the U.S., ophthalmologists rarely specialize in vision rehabilitation. However, the American Academy of Ophthalmology does recognize and promote vision rehabilitation (https://www.aao.org/low-vision-and-vision-rehab). Neuro-ophthalmologists are highly trained specialists with board certification in ophthalmology and/or neurology and they are often asked to evaluate IRF patients, usually after discharge. However, timely access to these highly trained specialists can be challenging, and their emphasis is not always on vision rehabilitation.

Despite the high prevalence of visual impairments in IRFs and the role of the OT to address related impairments, integrated services are limited to the OT scope of practice including task or environmental modifications, education, or oculomotor activities/exercises. OTs are not trained to provide diagnosis of the visual system in order to deliver interventions tailored to the patient's visual system diagnosis or provision of prisms for diplopia or hemianopic field expansion. Therefore, it would be beneficial to collaborate with an OD specializing in vision rehabilitation to create a more comprehensive care pathway directly in the IRF setting. Some precedent does exist for a more robust inpatient eye and vision assessment and rehabilitation program both in the U.S. Veterans Administration System at Polytrauma Network Sites (eg, Palo Alto), and as described in the U.K. via orthoptists who perform vision assessment and prism application.^7^^,^^15^^,^^16^ A high spontaneous recovery rate of OCNP may be cited as a reason to withhold early intervention. While some forms of OCNPs can resolve completely (eg, caused by demyelination or peripheral diabetic ischemia), those caused by stroke, aneurysm, and traumatic brain injury are often more severe and persistent.^17^ Chalouhi et al reported only 37.8% complete recovery of third nerve palsies at about 12 months after endovascular treatment for posterior communicating artery aneurysm (n=37).^18^ Complete recovery of traumatic bilateral sixth nerve palsy cases was only 27%.^19^ The prognosis for hemianopia is even worse, with a complete recovery rate of only 4% at 6 months.^20^ While hemineglect typically shows substantial spontaneous improvement over time, it remains one of the main independent negative predictors of long-term rehabilitation outcome^21^^,^^22^ with functionally significant long-term deficits^22^, ^23^, ^24^, ^25^, ^26^, ^27^, ^28^ even in patients who have no obvious signs on general physical and ophthalmologic examination.^23^ In terms of reimbursement, while the Center for Medicare and Medicaid Services (CMS) dictates that for coverage of outpatient therapy services the underlying condition should not cause a transient and easily reversible loss or reduction in function.^29^ There is no such requirement for inpatient services. Rather, patients must be able to participate in, and benefit from therapy services for a tangible improvement^30^ which may be hampered by comorbid vision impairments.

The collaboration between OTs and ODs for neuro-rehabilitation care carries potential benefits by leveraging the OD's expertise in the provision of ocular and visual system assessment and optical and diagnosis-specific compensatory and oculomotor therapeutic interventions, and the OT's expertise in addressing the effect of the visual impairments on patients’ physical, social, emotional well-being, and overall safety. In the special case of inpatient acute rehabilitation where an OT is interacting with the patient on a daily basis, there is a unique opportunity for the OT to reinforce the OD recommended diagnosis-specific and optical interventions, if the expectations are feasible for the OT. Such a collaboration, if successful, should improve patient outcomes.

This study investigated the clinical development, implementation, and 5-year retrospective study of collaborative OT-OD visual neuro-rehabilitation care pathway for inpatient vision assessment and rehabilitation using diagnosis-specific protocols across 2 IRFs and 1 Long-Term Acute Care Hospital (LTACH) within a major U.S. rehabilitation network. The implementation process involved workflow development for screening and identifying visual problems, OD specialist evaluation and diagnosis, and provision of vision rehabilitation interventions by the OD and primary treating OT. The rationale for the interventions selected and the feasibility of implementation are also reported. We anticipated that the most commonly referred visual conditions would match those targeted with the diagnosis-specific protocols. Secondarily, OT utilization, feasibility, and perceived value were measured with the hypothesis that the service was feasible for the OTs to deliver. For the purposes of this study, feasibility for the OT would be defined by clearly specified protocols within their scope of practice for which they received adequate training and support and which could be accomplished by most patients in the time available during their typical 30 to 60-minute daily treatment sessions.

## Methods

### Study design and setting

This was a retrospective study of a clinical program involving 2 IRFs (referred to here as IRF-1 and IRF-2) and 1 LTACH. IRF-1 is a 132-bed acute care facility in an urban area with an average length of stay of 2-4 weeks. IRF-2 is a 60-bed acute care facility a minimum of 1-hour drive from the nearest major metropolitan area with an average length of stay of about 2 weeks, and the LTACH is an urban 180 bed long-term acute care facility with an average length of stay of >25 days. The retrospective research protocol was approved by our institutional review board human subjects committee and conducted in accordance with the tenets of the Declaration of Helsinki. The institutional review board determined that informed consent was not required, given the retrospective nature of the study. The clinical process is described along with the retrospective research methods in sufficient detail to allow replication.

#### Pre-existing state of care

Prior to the implementation, the LTACH had no eye or vision rehabilitation care. However, IRF-1 and 2 had an existing option to obtain eye and vision care consults by different OD or ophthalmologist providers at each site without coordination. A written protocol and training were available for OTs for conducting the patient initial visual skills screening. However, there were no written approved protocols, manual of procedures, or formal method of OT training for the vision rehabilitation component of care, and prisms were not provided by the IRF.

#### Vision service model

Leadership for the vision services at each site included an OT vision specialist appointed to handle communication with the OD, coordination of the clinic, and staff education. An initial meeting between the OD and OT leadership explored philosophies and scope of care leading to a shared mission and scope for the service. The program would expand upon similar protocols developed previously by the first author (K.H.) at another IRF, not named in this report. This included the capacity for a full OD assessment of the visual system including visual acuity, refractive error, ocular motility, binocular vision, visual fields, and ocular health (comprehensive eye exam as defined by U.S. CMS. Advanced diagnostic services such as optical coherence tomography,^31^ scanning laser ophthalmoscope,^32^ or automated computerized perimetry^33^ were not available onsite. Examples of equipment requested for the vision service included visual acuity charts for near and far,^a^ occluder paddles,^a^ retinoscope and lens rack (refractive exam),^b^ tonometer,^b^ hand-held slit lamp biomicroscope,^b^ ophthalmoscope.^b^ Diagnostic topical pharmaceutical ophthalmic solutions (eye drops) were provided by the hospitals for intraocular pressure measurement (Flurox or equivalent) and dilated ocular health examination (Tropicamide 1% or equivalent) with pharmacy orders placed by the attending medical staff the day prior to vision clinic. IRF-2 and LTACH provided a set of ∼15 mL dropper bottles labeled for each individual patient on the schedule (smallest size available without contracting a compounding pharmacy), while IRF-1 provided 1 ∼15 mL bottle of each diagnostic medication to be used with any patient where dilation or intraocular pressure measurement was determined to be necessary by the OD. Examples of equipment provided by the hospitals for visual rehabilitation interventions were Fresnel press-on prisms (∆; 4^∆^ and 8^∆^ for management of diplopia,^a^ and 40^∆^ Peli visual field expansion lens for hemianopia),^c^ prism adaptation goggles (for hemineglect),^a^ monocular eye patches,^a^ pre-made reading glasses, non-prescription (plano) glasses (for mounting prisms for patients without their own glasses), fixation sticks,^a^ and physiological diplopia cords (a vision therapy tool for binocular vision therapy),^a^ and plano (clear lens) eyeglasses.^d,e^ Cost for initial set up of the service was less than $7000 U.S. Dollars.

#### Clinical workflow

**Fig 1:**
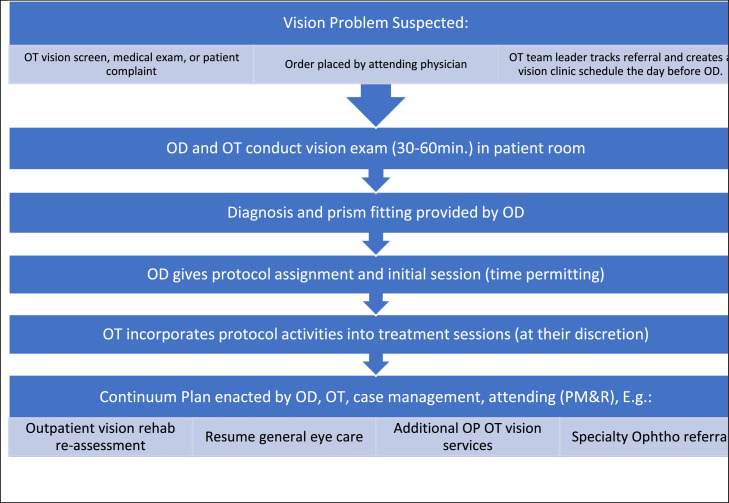
Inpatient vision rehabilitation care pathway.

Workflows differed slightly between sites, based on, local logistical needs, patient complexity, and availability of the OD staff. Clinic was provided either weekly (IRF-1), twice monthly (IRF-2), or monthly (LTACH). The process for vision clinic referral included physicians or therapists identifying patients who may derive benefit from consultation with the onsite OD (fig 1). From that point, attending physicians signed orders in the electronic medical record which were forwarded to the OT clinic coordinator. The triaging of urgency for the consultations was typically decided upon by the OTs, at times, in conjunction with the physician if there were pressing eye health concerns. Once the referral was made, it typically took about 3-5 days at IRF-1, and possibly longer at IRF-2 and LTACH, for the patient to be seen depending on urgency and timing. The diagnostic pharmaceuticals were dispensed by the pharmacy directly to the ODs or the OT coordinator immediately prior to each clinic and returned to the pharmacy afterward for tracking and disposal or restocking (if unused). There were no instances of post diagnostic drop complications at any of the sites.

Vision rehabilitation protocols were developed for the most common vision conditions. Based on available literature, homonymous hemianopia, third, fourth, and sixth cranial nerve palsies, and left hemispatial neglect were anticipated to be the most commonly encountered visual problems. Additionally, a protocol for cortical blindness (ie, bilateral hemianopia) was requested by the OT leadership. The OD produced draft protocols for these impairments which were reviewed by the OT vision specialist at each site for content and feasibility. Additionally, 2 general protocols were added for binocular vision and oculomotor problems which did not fit into the diagnosis-specific protocols. The OD and OT leadership reviewed protocol drafts prior to any training or implementation with an opportunity for discussion to modify any procedures. The OD would ideally, for logistical and budgetary staffing reasons, perform the consult during the general OT treatment session, provide diagnosis, and usually an initial session of vision rehabilitation as defined in the corresponding protocol (appendix 1). The OT would then reinforce the protocol activities at the recommended frequency during their typical sessions and contact the OD as needed. Follow-ups were not routinely conducted by the OD, unless deemed necessary, because of the high demand for new consults.

#### OT staff training

Initial training was in person, and a video recording was uploaded to a shared secure folder on the institutional network for onboarding of new OTs and as a refresher course for existing OTs as needed. This shared resource also stored the written vision treatment protocols (appendix 2) and a detailed manual of procedures (supplement).

#### Special interest group methods

Meetings were scheduled on an approximately monthly basis from 2015 to 2017, with participation offered both by institutional email and word of mouth. Group members were not required to be present at every meeting to contribute and would attend as their schedules allowed. Tele-conferencing was used to facilitate collaboration and attendance between sites, and a rotating schedule was used such that each site had an opportunity to host the in-person meeting at least once. Topics included patching (monocular occlusion) vs press-on prisms for diplopia, postural adaptations for paralytic strabismus (third, fourth, sixth nerve palsies), disuse in paralytic strabismus, oculomotor re-education activities, as well as the rationale for or against their use. The literature available was reviewed and discussed for any guiding value for the specific protocols (see appendix 1, and manual of procedures in a supplement). No formal consensus methodology was used in reviewing the protocols and responses during the special interest group (SIG) meetings were not anonymized (OT survey responses described later were anonymous). This process was feasible and typical for the development of such pathways and protocols within the rehabilitation network. Effort was made to elicit responses and feedback from participants by the SIG mediators (authors K.H., K.S., M.K., and R.T.).

#### Participants

A median of 36 occupational therapists (OT) and 2 low vision rehabilitation optometrists (OD) participated in the SIG meetings.

#### Retrospective record reviews

Five years after implementation of the inpatient vision rehabilitation care pathway, the billing database was queried for all vision clinic encounters from October 1, 2014, through December 31, 2019, to obtain counts of patients seen by the ODs, diagnoses, and available demographics of age and sex (race/ethnicity not available), see table 1. Multiple encounters or procedures on the same day (eg, consult visit and sensorimotor exam) were filtered to prevent duplicate counts. Additionally, the IRF Electronic Data Warehouse (EDW) was queried for all notes filed by the 2 OD providers for all 3 sites January 2017-December 2019 (unlike the billing database, which provided data for the entire 2015-2019 timespan). 2017 was the first full year in the EDW because of a new electronic medical record^f^ implemented in 2016. EDW data contained the date and time the consult note was filed, provider name, and service (stroke, brain injury, etc) but diagnoses and demographics were not available in the EDW. A second more detailed query was performed matching the EDW data to patient note detail from Epic Workbench which contained demographic data. This process provided age, sex, and ethnicity data, but visual diagnosis was only 1 of up to 40 diagnoses under the hospital admission and so was not able to be extracted reliably, within the funding constraints of the project (table 2). Additionally, counts of total admissions at each site were tabulated from Epic SlicerDicer, for available years of 2018 and 2019.

**Table 1:** Five year review of diagnoses from Vision Service Billing Database

Diagnosis	Third	Fourth	Sixth	INO	VGP	CGP	OMCNP	VS	CI	
**Patients seen (%)**	**79**	**35**	**122**	**22**	**32**	**57**	**347**	**131**	**144**	
	**(3.8%)**	(1.7%)	**(5.9%)**	**(1.1%)**	**(1.5%)**	**(2.7%)**	**(16.7%)**	**(6.3%)**	**(7.0%)**	
Age (y) Mean ± SD	49 (21)	50 (26)	49 (21)	58 (18)	53 (20)	58 (17)	50 (22)	57 (23)	63 (22)	
Sex (% women) –	48%	47%	52%	41%	63%	47%	51%	48%	47%	
**Diagnosis (Continued)**	HH all	RHH	LHH	HSN	HSN+L HH	HSN+ RHH	XK	Nystag	CVI	**Total**
**Patients seen (%)**	**386 (18.5%)**	**136 (6.5%)**	**181 (8.7%)**	**399 (19.2%)**	**97 (4.7%)**	**13 (<1%)**	**34 (1.6%)**	**94 (4.5%)**	**8 (<1%)**	**2083 (100%)**
Age (y) Mean ± SD	67 (20)	62 (21)	69 (15)	69 (18)	71 (10)	55 (23)	49 (18)	54 (20)	56 (22)	59 (21)
Sex (% women)	49%	45%	42%	51%	36%	43%	52%	57%	40%	44%

Abbreviations: CGP: conjugate gaze palsy; CI: convergence insufficiency; CVI: cortical visual impairment; HH: homonymous hemianopia; HSN: hemispatial neglect; INO: internuclear ophthalmoplegia; LHH: left homonymous hemianopia; OMCNP: oculomotor cranial nerve palsy; VGP: vertical gaze palsy; VS: vertical strabismus; XK: exposure keratopathy.

**Table 2:** Brain injury service sample from EDW review, January 2017-December 20

Ethnic Category	Women	Men	Total
Hispanic or Latino	5 (1.7%)	7 (2.4%)	12 (4.1%)
Not Hispanic or Latino	101 (34.5%)	180 (61.4%)	281 (95.9%)
**Ethnic category: total of all subjects**	106 (36.2%)	187 (63.8%)	293 (100%)
**Racial Categories**			
American Indian/Alaska Native	0	0	0
Asian	11 (3.8%)	10 (3.4%)	21 (7.2%)
Native Hawaiian or Other Pacific Islander	0	0	0
Black or African American	4 (1.4%)	8 (2.7%)	12 (4.1%)
White	10 (3.4%)	22 (7.5%)	32 (10.9%)
**Racial categories: total of all subjects**	25 (8.5%)	40 (13.7%)	65 (22.2%)

#### Anonymous OT survey

As part of a clinical quality improvement process, a 10-item survey was developed and distributed by paper and electronically via email and online using Survey Monkey (see appendix 2). The retrospectively acquired survey data were further analyzed as part of the study but were not originally designed for research purposes. As such, the data had not been validated, which is common with such clinical quality improvement efforts. All OTs on the inpatient units, as well as a few select OTs who participated in the SIG and formerly worked on the inpatient unit, were invited to participate. The survey questions were in the form of categorical or Likert-type responses, or multiple-choice range bins (see the entire survey available in the appendix). Item response choices used terminology recommended by the survey generation software, representing accepted methodology from previously validated instruments. A *review-and-revise* method was used among the authors to select question topics and optimize language clarity.

#### Primary outcome measure

Vision service utilization via tabulation of consults provided and diagnoses.

#### Secondary outcomes

Anonymous OT survey summary statistics, gaps in intervention/training (ie, identification of high frequency diagnoses without developed protocol[s]), differences in responses for the LTACH and IRFs.

#### Statistical methods

Summary statistics were calculated for median and 25th-75th interquartile ranges and frequencies were tabulated for categorical outcomes, reported as counts or percentages. For the survey analyses, binned range data items were collected (eg, 0%-25% of the time, 25%-50% of the time, etc). For these bins, the midpoint was reported for the median bin (eg, 25%-50% bin was reported as 38%). Interquartile ranges (IQR) were converted based on the proportion of the IQR bin (eg, if bin=0 to 25%=1, then if 25th%=1.25, value reported=25*.25=6.25). Statistical comparisons were performed including non-parametric analyses (Kruskall-Wallis test) to compare the anonymous survey responses, and a Wilcoxon Rank-sum test for pairwise comparisons of LTACH and acute IRF responses. Correlation analyses were conducted using Pearson's or Spearman's test as appropriate following verification of data distribution.

## Results

### Vision special interest group results

The SIG reviewed and approved the protocols based on the rationale and available evidence described in detail in appendix 1.

#### OD role

The OD visual neuro-rehabilitation specialist's role was to provide ocular and visual system assessment, diagnosis, and assignment of a vision rehabilitation protocol when appropriate. An assessment report included these aforementioned components, addressed other ocular issues, provided triage recommendation for urgent issues, and supported a continuum of eye and vision rehabilitation care plan. The OD would usually conduct an initial session of the vision rehabilitation protocol with the patient and OT, reviewing concepts and answering questions.

#### OT role

The role of the OT included conducting the initial vision screening (appendix 3) and recommending referral for further visual evaluation when appropriate. The treating OT (IRF-1) or OT vision specialist (IRF-2 and LTACH) would attend the vision clinic appointment. Afterward, the OT role was to reinforce educational components and appropriate use of assistive devices (eg, prisms), evaluate and make recommendations concerning safety and response to adaptations and devices, and perform oculomotor re-education activities with the goal of the patient being able to self-administer the activities by the time of discharge. The OT was responsible for reviewing the written OD report in the patient's medical record, and providing team communication concerning visual diagnoses and care plan (OD was not asked to attend interdisciplinary team meetings). The OT was responsible to reach out to the OD with any questions, concerns or changes especially around use of prisms and Peli lenses.

#### Diagnosis specific protocols

##### Protocols for the rehabilitation of ocular cranial nerve palsies

**Fig 2:**
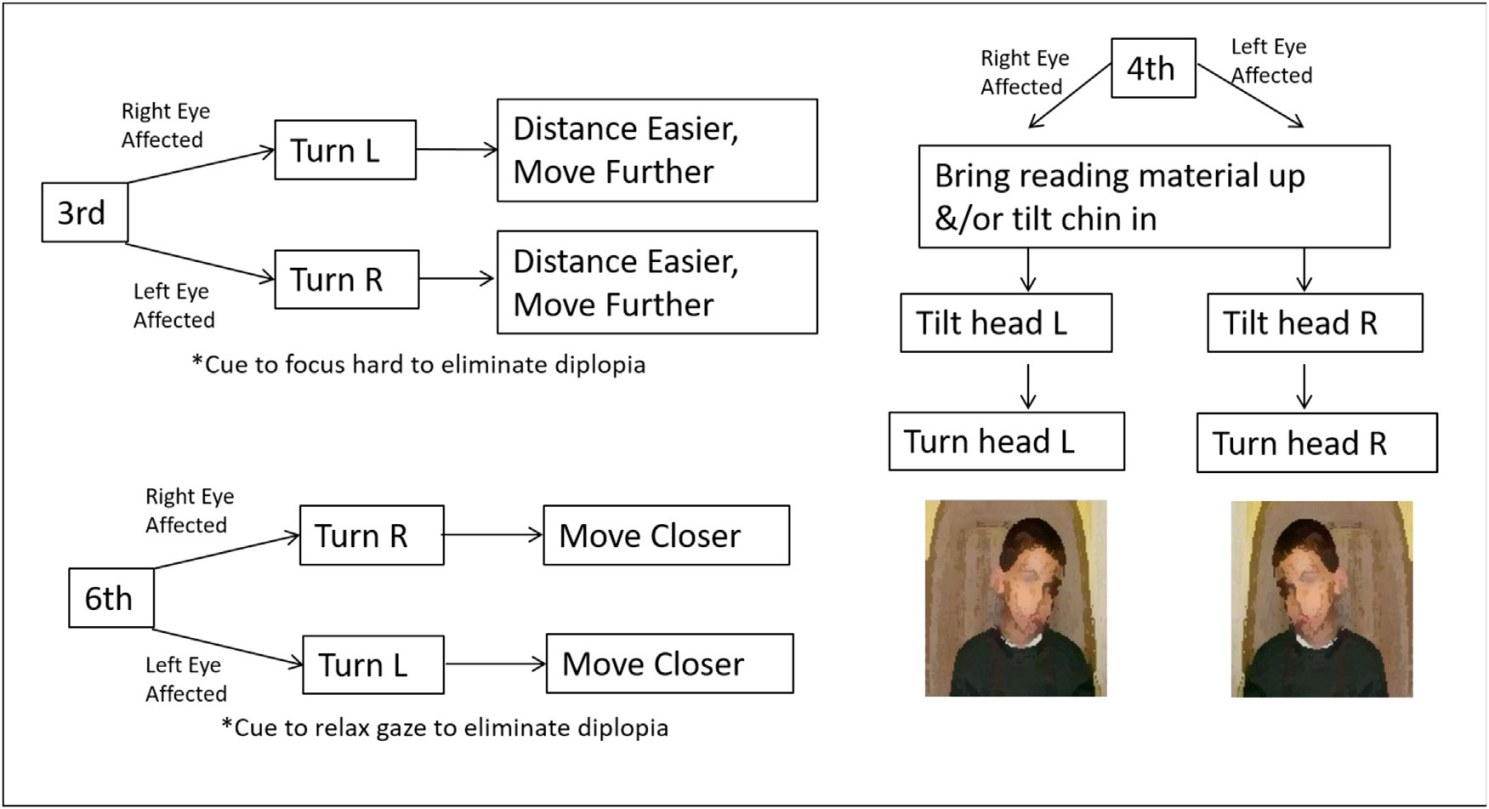
Compensatory postural and environmental modifications flowchart to reduce degree of strabismus. This chart was presented as part of the training and made available to OTs for clinical reference.

OCNPs composed of third, fourth, and sixth nerve palsy protocols are discussed together as they are based on similar principles with the only differences being the directionality of compensatory posturing and exercise. The detailed protocols are available in the appendix 3 along with justification and supporting literature citations in appendix 1. To summarize, the first component of these protocols was postural and environmental modifications, which involved cueing the patient to perform a head-turn, tilt or adjust the task space to move the desired point of gaze away from the paretic motor field where the angle of strabismus was either minimized or eliminated (fig 2).

**Fig 3:**
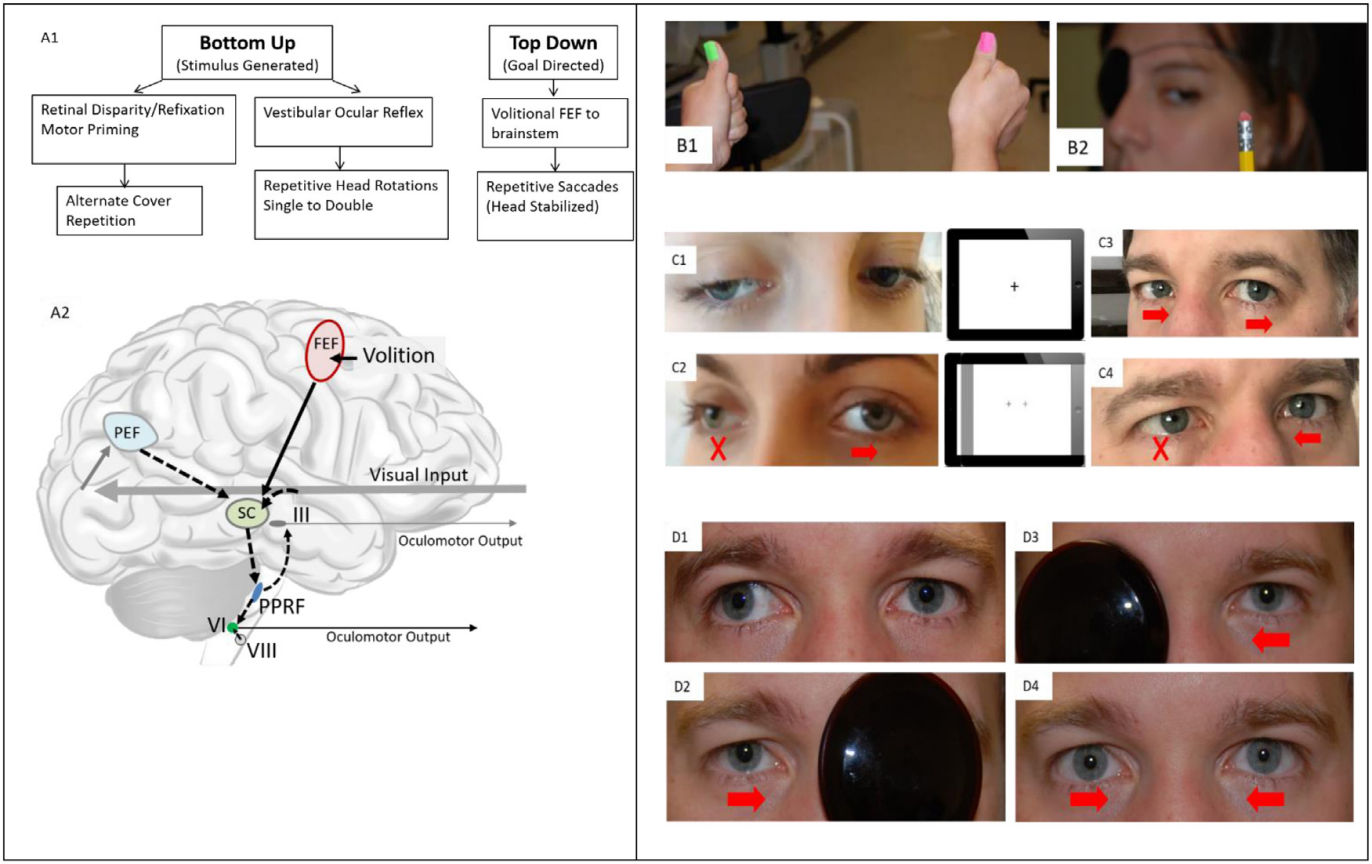
(A.1) Repetitive oculomotor neuro re-education protocol activities were selected to use the main oculomotor pathways. (A.2) Oculomotor pathways in the human brain, simplified. (B) Top-down (Volitional) activities, including repetitive saccades in B.1 and pursuits in B.2, originate in the frontal cortical networks. (C.1 and 2) Head rotation activity illustrated for right third nerve palsy, characterized by an inability to fully adduct and supraduct the right eye. (C.3 and 4) Head rotation activity illustrated for right sixth nerve palsy, characterized by an inability to fully abduct the right eye. Note that the activity is the same as for third nerve but in the opposite direction. (D) Photo-illustration of the alternate cover activity. (D.1) There is exotropia of the right eye similar to that occurring in right third nerve palsy, and the left eye is fixating a penlight (note corneal reflection). (D.2) Upon covering the left eye, the exotropic right eye reflexively saccades inward to fixate the penlight via the PEF to SC pathway. Under the cover, because of the law of equal innervation, the left eye is now exotropic. (D.3) Upon alternating the cover, the left eye saccades inward to re-establish fixation. Under the cover, the right eye returns to an exotropic posture. This alternation is repeated approximately 10 times as tolerated while stabilizing the head to prevent avoidance posturing. (D.4) After the alternation cycles are completed, the cover is removed. The patient may spontaneously converge to align the eyes as shown in the photo. Alternatively, they may need to be cued to “try to bring the double images together.” The process was repeated up to 5 times per session, as tolerated. Abbreviations: PEF, parietal eye field; FEF, frontal eye field; SC, superior colliculus; PPRF, paramedian pontine reticular formation; III, oculomotor nucleus; VI, abducens nucleus; VIII, vestibular nucleus (VOR).

The second OCNP protocol item was the use of press-on Fresnel prisms (3M, St. Paul, MN), either 4^∆^ or 8^∆^ (only these strengths were stocked, as our prior experience suggested that this would be sufficient for most patients, see appendix section 1 for detailed discussion and references). The protocol advised cooperative use of the prism and postural adaptations. Monocular occlusion (patching) would only be used in cases where prisms and/or postural modifications did not allow safe and adequate functioning. The third protocol item was oculomotor neuro re-education, a historical term used for physical medicine and occupational therapy by the CMS to describe defined roles in the practice scope of the OT. Oculomotor neuro re-education aimed to reduce antagonist contracture, discourage disuse atrophy, promote recruitment of nearby musculature, promote uni- and bino-saccadic and vergence adaptation, and improve vergence ranges using current understanding of eye movement neurophysiology by targeting the available eye movement neural pathways (fig 3). This included repetitive large amplitude and velocity saccades (eye jumps), pursuits (tracking), head rotations (with fixation on a stationary target), repetitive alternate cover un-cover (reflexive saccades and vergence), and vergence and accommodative therapy (ie, alternate cover un-cover, pencil push-ups, Brock string). For detailed rationale and supporting literature, see appendix 1. A copy of the implemented protocols is provided in appendix 2.

##### Homonymous hemianopia rehabilitation

Evaluation for hemianopia with the ODs was offered in cases where there was suspected field loss. The hemianopia vision rehabilitation protocol was typically offered when the OT indicated the field loss was affecting activities of daily living (ADLs). If the OT was unavailable or unsure, the OD considered the severity of hemianopia, awareness of the visual loss, and the demonstration of compensatory behaviors such as the ability to quickly locate peripherally placed objects. Differentiating hemianopia from lateralized attentional deficits related to hemineglect was a common request by the OT staff.

##### Hemianopia rehabilitation protocol

**Fig 4:**
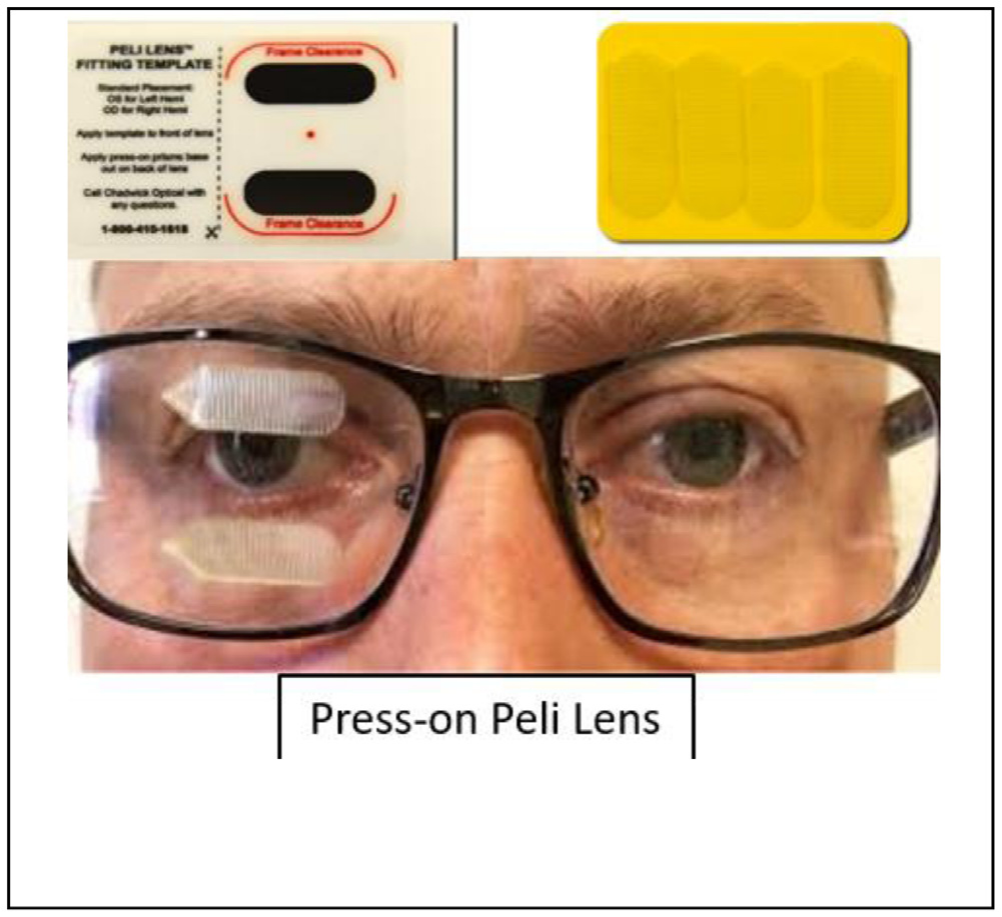
40 prism diopter (18°) Peli lens, standard fitting for right hemianopia, available in the U.S. exclusively via Chadwick Optical, Harleysville, PA.

A copy of the SIG-approved Hemianopia protocol is available in appendix 1 along with justification and supporting literature citations. To summarize, the hemianopia protocol firstly included patient education by the OD, with explanation and demonstration to improve awareness of the field loss, demonstration of the retinotopic nature (the fact that field loss moves with gaze shifts), and eccentric viewing training (dissociating attention from fixation, ie, gazing to the side but attending centrally). Next, fitting of Peli lenses for field expansion was performed by the OD when visual field loss was grossly complete and any residual vision in the affected field was not better than hand-motion without projection (fig 4). The OD provided training on Peli lens use according to manufacturer's instructions (Chadwick Optical, Harleysville, PA). Afterward, the OT reinforced education and Peli lens concepts, performed safety monitoring continuously during therapy sessions, conducted compensatory scanning training via cueing during ADL training (or less commonly using scanning light boards or saccadic workbooks/apps), and provided training on the selection and use of visual anchors (such as drawing of a red line in the margin) with the goal of improving systematic visual search. Reading rehabilitation was also conducted by the OT when the OD provided a diagnosis of hemianopic dyslexia, via training on the selection of page anchors and the use of line guides.

##### Prism adaptation therapy protocol for left hemispatial neglect

A copy of the detailed SIG-approved prism adaptation protocol is available in appendix 1 along with justification and supporting literature citations, including discussion of the multiple randomized controlled trials and meta-analyses. To summarize, prism adaptation therapy (PAT) was performed similar to that described originally by Rossetti et al^34^; however, because of supplier availability, rather than the 10° rightward deviating prisms (17.6^∆^) described by Rossetti, we used 20^∆^ base left (11.4° rightward deviating) goggles (Bernell VTP Inc, Mishiwaka IN), similar to those described in the Kessler Neglect Assessment Process and available through their website (West Orange, NJ).

#### Retrospective review results

Billing data base results: In the 5 years during which the above-described protocols were implemented and conducted, there were 2083 unique patient billing encounters. As predicted, the most common diagnoses were hemispatial neglect (n=399, 19.2%), homonymous field defects (n=386, 18.5%), and oculomotor cranial nerve palsies (OCNPs (n=347, 16.7%). Further breakdown is available in table 1. Notably, there were 144 cases of convergence insufficiency (7.0%) and 94 cases of nystagmus (4.5%) which did not have a diagnosis-specific protocol. There were 8640 total admissions across the 3 sites in 2018 and 2019, of which 9.6% (n=833) were seen by the vision service.

#### Anonymous OT survey results

All OTs working on the inpatient units (n=66) were invited to participate. Of these, there were 52 full time staff, 4 floats, and 10 practice leaders. It was estimated by the practice leaders (authors T.M., K.S., and M.K.) that only about half of the staff would be administering the protocols on a regular basis, with the others either not carrying caseloads or working on units that did not request vision consults regularly. These OTs would have been unlikely to respond to the survey. Therefore, it was expected that about 33 OTs would respond. Reminders were sent out approximately weekly until the response rate was near the expected 33 OTs. Thirty-one OT responses were received (94%); 21 of whom reported working in 1 of the acute IRFs, 8 in the LTACH, 1 in outpatient (formerly worked at IRF-1), and 1 other (location unknown, not specified). Twelve out of 31 OTs had 5 years or more of experience, and so would have been present for most of the period of the record review. None of the respondents were excluded in our reporting.

**Fig 5:**
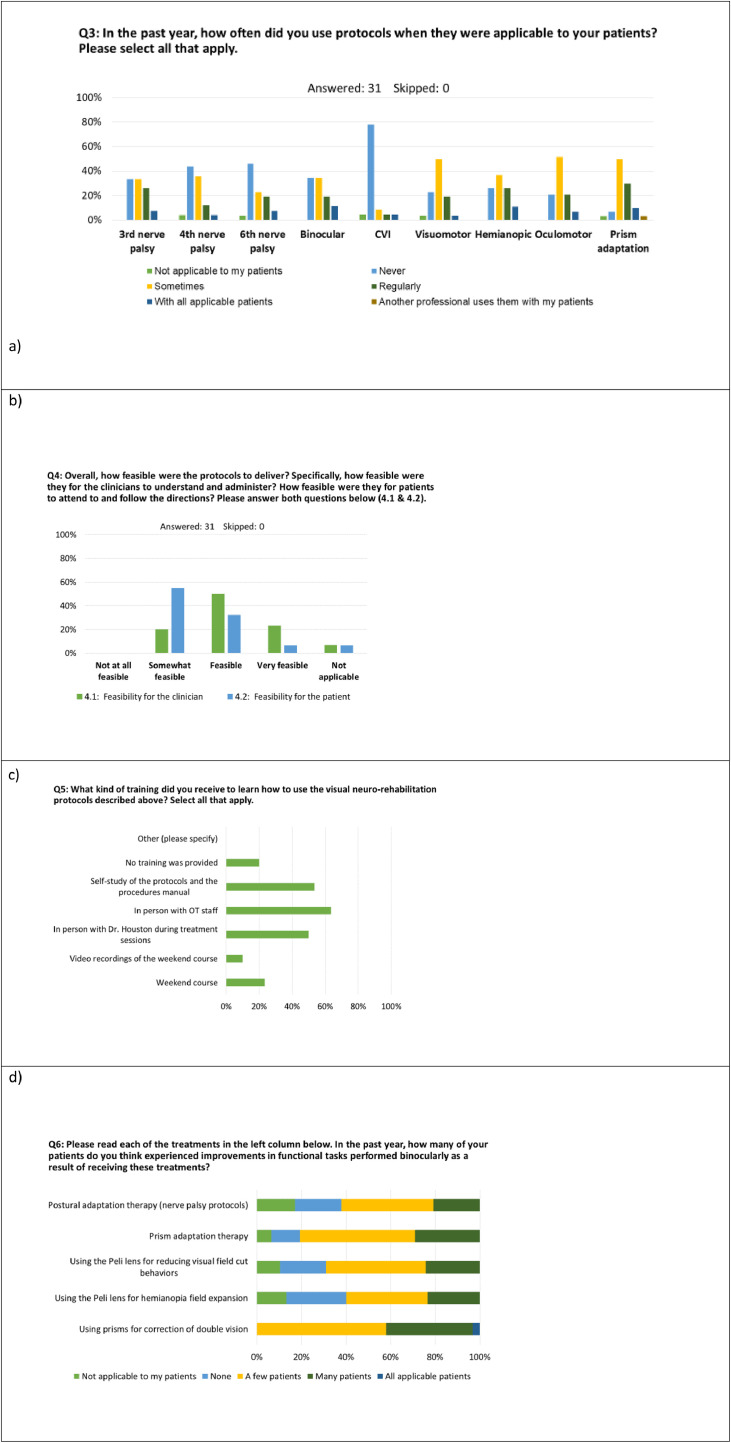

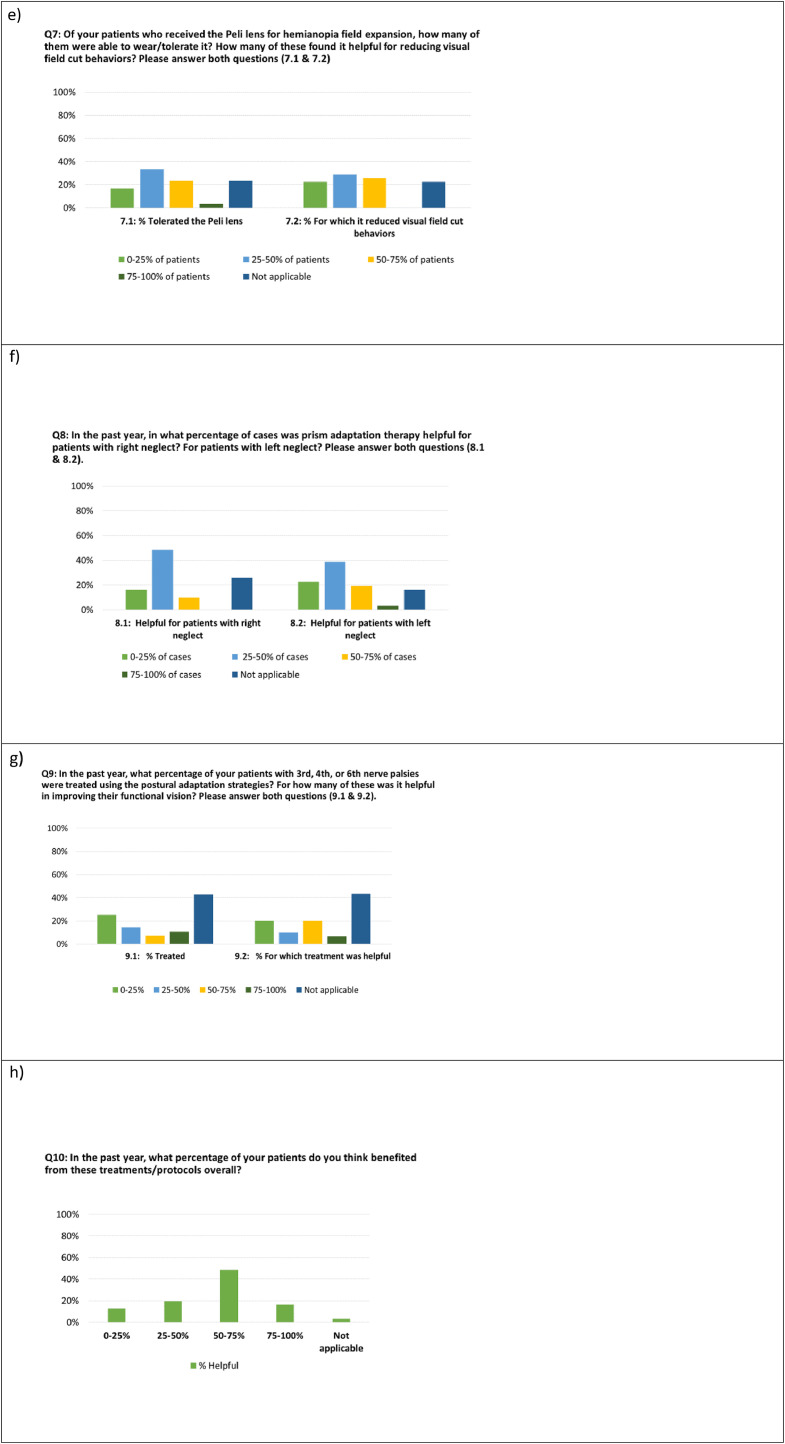
Bar plots of anonymous OT survey responses.

Overall, the OT respondents indicated that the protocols were feasible for them to deliver (most common response was “*feasible”* [50%, n=15]), fig 5b. The OTs most often reported that the protocols were *somewhat-feasible* for their patients (n=17), with the second most common response being *feasible* (n=10). The “*Not-at-all-feasible for patients*” option was not selected by any OTs. OTs indicated that in the past year 63% (IQR 38%-69%) of their patients benefited from the protocols, with no significant difference between LTACH and acute settings, *P*=.40. For the nerve palsies (third, fourth, sixth), 42%-58% of respondents used the protocols sometimes or more regularly, whereas 29%-39% of OTs never used any of the nerve palsy protocols (fig 5b). There was no difference in reported frequency of use by nerve palsy type (χ^2^=2.0, *P*=.40). OTs were specifically asked about their use of the postural modifications component of the nerve palsy protocols, with only 10% of OTs providing this particular intervention 75% of the time or more (fig 5g left panel, blue bar). All OTs reported that the press-on Fresnel prisms for nerve palsies provided benefit for at least some of their patients with 55% indicating the prism helped a few and 42% indicated that it helped many or all (fig 5d). For the Hemianopia Protocol, there was no significant difference in OT reported usage as compared with the Nerve Palsy Protocols, (χ^2^=4.6, *P*=.20), with an equal number (n=10) of the *sometimes* and *regularly* to *always* response options (fig 5a). For the Peli lens (hemianopia field expansion), OT responses suggested a median patient tolerance of 38% (IQR 19%-68%), similar to prior studies in outpatient populations.^14^^,^^35^ Of those who were able to tolerate the Peli lens, OTs reported a median of 38% (25%-68%) of those patients had “noticeably reduced field cut behaviors”. For the PAT protocol for left hemineglect, reported usage was either *sometimes* or *regularly* for 77% of responses. A median of 38% (25%-38%) of patients had noticeably lessened signs of left neglect attributed to the treatment (fig 5f). Some OTs rated PAT for right neglect (although the treatment was never prescribed for right sided neglect), suggesting there may have been some confusion surrounding indication (table 3). These questions were designed to test OT understanding of indications and appropriate diagnosis-specific use in order to determine if education efforts were effective or if additional training may be needed, (see appendix 2, OT survey question 8). Other signs of protocol misunderstanding were minimal but included reporting the use of Peli lens for non-field related disorders (n=3), vergence training or prism for cortical blindness (n=2), and vergence training for visual field loss (n=1). No association with training type or years-experience was evident on visual inspection of data, and so further analysis was not indicated.

**Table 3:** OT survey report of vision rehabilitation interventions used by visual impairment type

	Visual Field Problem	Visual Neglect	Cortical Visual Impairment (CVI)	Eye Movement Disorder/Diplopia	Total Respondents
**Field expanding prisms (Peli)**	90.00% 18	70.00% 14	5.00% 1	10.00% 2	20
**Other prism**	53.85%	46.15%	7.69%	69.23%	
	7	6	1	9	13
**Postural adaptation**	25.00%	25.00%	41.67%	50.00%	
	3	3	5	6	12
**Scanning (non-computerized)**	90.00% 18	95.00% 19	25.00% 5	35.00% 7	20
**Scanning (computerized)**	90.00% 9	90.00% 9	30.00% 3	30.00% 3	10
**Field enlargement training**	90.00% 9	40.00% 4	0.00% 0	10.00% 1	10
**Vergence exercises**	5.88%	0.00%	5.88%	100.00%	
	1	0	1	17	17
**Training for saccades, pursuits, fixation**	31.58% 6	26.32% 5	42.11% 8	84.21% 16	19
**Environmental modifications**	100.00% 19	100.00% 19	57.89% 11	52.63% 10	19
**ADL training**	91.30%	100.00%	82.61%	82.61%	
	21	23	19	19	23
**Assistive devices**	50.00%	50.00%	100.00%	50.00%	
	3	3	6	3	6
**Providing explanations/information**	100.00% 19	100.00% 19	84.21% 16	94.74% 18	19
**Not applicable**	0.00%	0.00%	0.00%	0.00%	
	0	0	0	0	0

NOTE. Highlighted data show interventions that seemed to be inappropriate for the designated visual problem and may represent areas where additional training is needed**.**

Abbreviation: CVI, cortical visual impairment.

## Discussion

### Main findings

This study investigated the development, implementation, and evaluation of a coordinated OT-OD collaborative multi-site care pathway for inpatient vision assessment and rehabilitation using diagnosis-specific protocols. Over the 5-year period, 2083 vision consults were provided across the 3 different inpatient sites including 2 acute IRFs and 1 LTACH. As anticipated, the most common visual impairments referred to the vision service were hemineglect, homonymous hemianopia, and oculomotor cranial nerve palsies (in that order), ranging from 14% to 17% (table 1), and together representing most of the cases (∼55%). This supports our targeting of these disorders with the protocols as developed. Caution should be taken in comparing these proportions to the same conditions in prior studies designed to determine prevalence in stroke or brain injury populations, such as those cited in the introduction.^2^, ^3^, ^4^, ^5^, ^6^, ^7^ Our sample is drawn from all inpatient units and only represents the portion of the inpatient population referred for vision assessment. Our sample would be expected to have a greater proportion with substantial visual impairments when compared with those studies cited in the introduction which evaluated all admissions. Given the broad sources of patient referrals, our sample is likely to be more representative of the possible scope of service that can be provided. Because not all patients could be evaluated because of the limited availability of the ODs, the OT service directors were sometimes forced to prioritize scheduling based on the teams’ judgment of the patient's need and the ability to participate and benefit. The EpicSlicerDicer reviewed from 2018 to 2019 suggests only about 10% of the entire admitted population was evaluated. There were no strict criteria for prioritization of consults, but some examples included suspicion of visual sequela, perceived influence of visual issues on ADLs, planned length of stay, patient/family anxiety level about the visual disturbance, and likelihood to be able to participate in vision testing. Additionally, when the primary OT indicated the patient needed the OT present to assist with participation in the examination, the clinic coordinator modified the schedule to make that accommodation. The counts reported here represent a valid estimate of the volume that similarly sized inpatient facilities could expect to have, although a full-time service to evaluate all patients would be warranted given the high prevalence of visual problems and reduced symptom reporting in this population. For logistical and budgetary reasons, the OT leadership selected an approach of training the primary treating OTs to provide this care during the daily OT sessions, as opposed to adding treatment sessions with an OT-vision specialist. As needed, OT vision service directors co-treated with the patients’ primary OT to reinforce the approaches outlined in the protocols. Use of a dedicated OT-vision staff is another approach that might be used to enhance service delivery.

The findings from the OT survey support that the protocols were feasible to deliver for 80% of OTs and beneficial to most of the patients (63%), even in the LTACH where patients were lower level (see Anonymous OT survey results section). Data from the OT questionnaire were obtained anonymously, minimizing bias as much as possible by encouraging candid responses. It was not expected that the protocols would be feasible for all patients, a situation which is common to any therapeutic intervention delivered on inpatient units. The 63% reported benefit was felt to be clinically acceptable, as evidenced by the volume of referrals over the 5-year period. None of the OTs reported the protocols were *never helpful or feasible* for their patients.

Prisms for strabismus were most often reported to help a few patients (55% of respondents), but nearly as many OTs reported they helped many or all (42%). Results for Peli lens (hemianopia field expansion) were similar with the most common OT response being that they helped a few of their patients, but more than 20% of OTs indicated many of their patients benefited. Unlike prisms for strabismus where no OT reported prisms were never helpful, there were ∼20% that reported the Peli lens never helped any of their patients with hemianopia. It should be emphasized that immediate tolerance of the Peli lens as applied during the OD exam was near 100%. The lens works based on scientifically well-understood and accepted optical principles and the field expansion is immediately measurable both by bedside confrontations fields and with more sophisticated perimetry measurements, see Apfelbaum et al, 2013 and appendix 1.2.^36^ The OT observations of the Peli lens represent an extended-use outcome, usually of about 1-2 weeks, and factored the ability of the patient to tolerate the lens (known to be difficult for about half of patients most often because of the difficulty interpreting the prism-shifted image ^14^) and the effects observed on daily tasks. While the survey items did not allow for an exact calculation of the perceived OT Peli lens success rate, we attempted to estimate this by binning the data, and arrived at 38% (see Anonymous OT survey results section), which is similar to 6-month follow-up previously reported in a double-blind randomized placebo controlled clinical trial.^14^ Interestingly, the level of tolerance vs benefit measured in Peli lens item 7 (fig 5) was nearly 1 to 1. This may suggest that those patients that are able tolerate the Peli lens mostly showed an improvement in their hemianopia behaviors, and therefore efforts to improve tolerance are logical. We think that improved tolerance can be best achieved via education of the hospital staff to understand how the patient is seeing and why they may have reduced awareness of their vision loss contributing to cases of intolerance (we only included OTs in our training). It could be argued that levels of reported success with relatively low cost (about $40 each) and with no major ill effects identified, warrants continued use. Studies which included objective outcome measures such as directly measuring frequency of gaze shifts to peripheral obstacles detected via the prisms, as done in some prior outpatient studies^37^^,^^38^ would be helpful to test the validity of the OT observations. Longer term studies examining outcomes of continued use, community re-entry, number of falls, and re-admissions are recommended for both Peli lens and strabismus prisms.

Prisms for hemineglect, that is, PAT, are quite different from hemianopia and strabismus. These prisms are used only during a short 5-10-minute session once daily and then not worn the rest of the time. A therapeutic benefit stems from a carry-over effect after the prism-aided visuomotor therapy task. The same prism goggles are used for all patients, and so the costs are essentially limited to the OT's time. Reports of benefit were similar to Peli lens, see figure 5, although a recent study suggested that untreated and treated patients may end up with the same outcome at the long-term follow-up even though the treatment may reduce the neglect initially relative to placebo groups.^39^ We note that these new results are based on meta-analyses which included variable between-study methodologies and inclusion criteria and should not be substituted for a proper sham-controlled RCT. A multi-center RCT is needed to address the conflicting data in the literature and appropriately guide clinical practice. There have been some efforts in this regard, including the development of a multi-center network led by Kessler Rehab in New Jersey (Network for Spatial Neglect, https://kesslerfoundation.org/researchcenter/stroke/nsnapplication).

### Study strengths and limitations

The current study employed methodologies to improve scientific rigor including masking of the author performing the primary statistical analysis, use of an anonymous OT questionnaire, and inclusion of survey items intended to test the respondents understanding of the indications for some of the interventions. However, while the survey used template language structure and scoring paradigms advised by the survey software, it has not been studied for internal and inter-rater reliability. This is a common limitation for such quality improvement surveys used for clinical purposes and therefore it is possible that the results do not accurately reflect the true observations of the respondents. Based on the number of surveys returned and the practice leader estimates of the OTs involved in using the protocols regularly being near 100%, it seems likely that the response rate from OTs regularly using the protocols was quite high. The survey responses indicated feasibility and benefits of the protocols while simultaneously demonstrating challenges with implementation. OTs’ utilization of the protocols did not always match the protocol's intention (eg, some respondents reported use of prism adaptation for right neglect and Peli lens for strabismus, see Anonymous OT survey results). Areas for improvement were noted as increased use of protocols for appropriate patients and proper protocol selection for visual impairment presentation. This could be related to their education, confidence, and continued competence with the protocols. While we acknowledge the survey limitations, to our knowledge, this study represents the first semi-quantitative data collection and analysis regarding such a program in the literature. The study conclusion of acceptable feasibility is also based on the continued referrals to the vision service providing care for more than 2000 patients during the 5-year period.

### Future directions

Based on the results of this review, we are considering some revisions and additions to these vision protocols. The protocols were created with the assumption of a single diagnosis and ability of the patient to participate. However, there were patients who were not appropriate for the created protocols because of their multiple nerve palsies, concurrent ocular visual impairments, or cognitive and behavioral deficits such as lethargy and delirium. This may have led to lack of or improper use of the protocols in some of these cases, which may have influenced the OTs’ responses. Some edits to the protocols are underway to improve the clarity of the inclusion and exclusion criteria and define acceptable modifications to protocol tasks. For example, internuclear ophthalmoplegia was fairly common, relative to other disorders in the nerve palsy category, but did not have a specific protocol. The ODs would often recommend use of the third nerve protocol activities because the pattern of strabismus is typically similar. A formalized internuclear ophthalmoplegia protocol is planned for implementation in the future. Comitant strabismus such as skew deviation and basic eso- and exotropia were also not uncommon. However, protocols were largely based on compensation (head posturing, task space) and repetitive exercise; none of which would be indicated for comitant cases. While our approach was to provide prism or monocular occlusion for these comitant cases based on techniques described in the Methods section OT role, generation of protocols specific to these strabismus types is not recommended or warranted.

Cortical blindness was uncommon (table 1) but given the profound effect on ADLs and interference with rehabilitation, it was helpful to provide some guidance for these difficult cases. It was also helpful to assign an OT-vision specialist to work with the general OT in these relatively rare cases.

Some changes to OT education procedures are planned based on the data obtained from this study, and additional informal feedback available. Development of an improved training module is underway providing better accessibility to recorded didactic educational material via upload to an internal website and addition of videos which demonstrate protocol administration. A pre-post-training knowledge assessment followed by return demonstration with OT vision service leadership was also recommended. There was highly variable use of the postural modification's component of the OCNPs which is being addressed by having the ODs provide the postural modification flowsheet directly to the treating OT with the diagnosis circled. In the absence of evidence-based practice guidelines, we have assembled a reasonable justification for the use of repetitive range-of-motion and vergence activities for OCNP (see appendix A1). Publication of this rationale allows the opportunity for review by other experts in the field, potentially leading to productive discussion and professional debate. We remain dedicated to delivering care which is consistent with modern understanding of oculomotor neuro-physiology, and open to changing the protocol materials based on feedback from the clinical scientific community.

The work presented here may be used as a foundation for creating or refining inpatient vision care pathways at similar IRFs. Uniform administration of vision neuro-rehabilitation care could provide a substrate for future clinical trials to evaluate efficacy.

## Conclusions

A multi-site care pathway was successfully developed and implemented for inpatient eye and vision rehabilitation care with evidence that hospital OT and PM&R staff perceived this program to be feasible and effective at a level acceptable clinically. This was evidenced by the high frequency of use of the service and supported by the survey results. Further studies are needed to optimize care delivery and determine efficacy.

## Suppliers

a. Visual acuity charts for near and far, Occluder paddles, Fresnel press-on prisms, Prism adaptation goggles, Monocular eye patches, Fixation sticks, Physiological diplopia cords; Bernell Corporation.
b. Retinoscope and lens rack, Tonometer, Hand-held slit lamp biomicroscope, Ophthalmoscope; Ophthalmic Instrument Co.
c. Peli visual field expansion lens; Chadwick Optical.
d. Plano (clear lens) eyeglasses; Fantas Eyes.
e. Plano (clear lens) eyeglasses; Cosmic Eyewear.
f. Workbench; Epic.

## Appendix Supplementary materials

Image, application 1

## Footnotes

Supplementary material associated with this article can be found, in the online version, at doi:10.1016/j.arrct.2022.100246.
