# Supplementary material for: Development and 5-year Evaluation of Diagnosis-Specific Protocols for Visual Neuro-Rehabilitation in a Multicenter Inpatient Rehabilitation Network
Source: Arch Rehabil Res Clin Transl. 2022 Nov 17;5(1):100246. doi: 10.1016/j.arrct.2022.100246 (PMC10036222; doi:10.1016/j.arrct.2022.100246)
Supplement: Supplementary file 1 [file mmc1.docx]

**Appendix**

**Appendix A1: Rationale for Protocol Activities with review of evidence base**

**A1.1 Protocols for the Rehabilitation of Ocular Cranial Nerve Palsies (OCNPs)**

OCNPs included 3^rd^, 4^th^, and 6^th^ nerve palsy protocols are discussed together as they are based on similar principles with the only differences being the directionality of compensatory posturing and exercise.

A1.1a) **The first component of these protocols was** **postural and environmental modifications**, which involved cueing the patient to perform a head-turn, tilt or adjust the task space to move the desired point of gaze away from the paretic motor field where the angle of strabismus was either minimized or eliminated (Figure 2). For example, in right 3^rd^ nerve palsy, strabismus is reduced as viewing distance is increased and shifted to the patient’s right (Figure 3C.1&2). Conversely, in right 6^th^ nerve palsy, the magnitude of strabismus is progressively reduced as the viewing distance is decreased and shifted to the patient’s left (Figure 3C.3&4). A postural flowsheet was available to the OT for review as needed on the institutional server (Figure 2), but if the patient was reliable in their report of diplopia, a trial-and-error process could be employed. The rationale for postural adaptation training was based on a well-known adaptive response to nerve palsies in children with congenital OCNPs.(Wright, Spiegel et al. 2013) In clinical practice, some adult IRF inpatients with strabismus had been observed to close an eye with variable frequency yet spontaneous adoption of one of these adaptive head or task space postures seemed to be less common. To promote this process, the OCNP protocols included a short daily session to repeatedly practice finding a head posture where a simple stimulus (a medium sized letter or symbol) was perceived as single, and then slowly moving towards the neutral position, stopping when double vision was perceived. The OT would then attempt to transfer this strategy during ADL/IADL (Activities of Daily Living/Instrumental Activities of Daily Living) training tasks for improved participation and performance. It remains unknown whether repeatedly cueing patients to employ such adaptations resulted in acquisition of this strategy and whether it resulted in better visual function, safety, and emotional well-being, although others have reported a similar approach.(Pollock, Hazelton et al. 2011) While determining efficacy was not the primary aim of this work, the OT’s were asked to document and quantify their observations. No objections were raised during the SIG meetings concerning the approach suggesting that efficacy was a reasonable assumption and that the cost-benefit was favorable as it was low risk, could be implemented during ADL training, and encumbered no equipment costs.

A1.1b) **The second protocol item was the use of press-on prisms** (3M, St. Paul, MN) fitted by the OD to reduce or eliminate the angle of optical axis misalignment caused by the OCNP. The protocol dictated cooperative use of the prism and postural adaptations. Use of prisms were supported by high reported success rates (Apers and Bierlaagh 1977, Flanders and Sarkis 1999, Tamhankar, Ying et al. 2011), efficiency of use and low cost (~$30 USD per prism).

Monocular occlusion (i.e., patching) is an alternative approach to managing acquired strabismus, with benefits being ease of application and little to no cost. The potential negative effects of patching that were considered included loss of ~20° of the binocular visual field (the far peripheral field is only seen by one eye), a more than 2-fold loss of visual sensitivity (phenomenon of binocular summation),(Schwartz 2010) potential negative effects on recovery due to a reduced sensory stimulus for fusional vergence eye movements, interference of uni- and bi-ocular saccadic adaptation mechanisms,(King 2011) evidence for functional visual impairment caused by monocular vision,(Ihrig 2013) and potential worsening of spatial neglect.(Houston and Barrett 2017)

A1.1c) **The third protocol item was oculomotor neuro re-education**. This terminology was selected based on historic use in physical medicine and occupational therapy by the Center for Medicare Services (CMS) to describe defined roles in the practice scope of the OT. Oculomotor neuro re-education aimed to reduce antagonist contracture, discourage disuse atrophy, promote recruitment of nearby musculature, promote uni- and bino- saccadic and vergence adaptation, and improve vergence ranges. This included repetitive large amplitude and velocity saccades (eye jumps), pursuits (tracking), head rotations (with fixation on a stationary target), repetitive alternate cover un-cover (reflexive saccades and vergence), and vergence and accommodative therapy (i.e. alternate cover un-cover, pencil push-ups, Brock string). Various combinations of these activities and instructions unique to each major diagnosis category were specified in the protocols (Appendix 2).

**A1.1c.1 Background and Rationale for Oculomotor Neuro Re-Education**

**Reduce antagonist contracture:** Contracture of antagonist musculature in OCNP is a well-recognized phenomenon,(Scott 1994) particularly in 6^th^ nerve palsy. Contracture is diagnosed by the presence of resistance on forced ductions test (Flanders, Qahtani et al. 2001), likely due to physical changes in muscle sarcomere length.(Scott 1994) For effective prevention of contracture, any treatment should occur prior to structural changes in the muscle, which can happen fairly rapidly after onset of OCNP. Botulinum toxin (“botox”) injection to the body of the medial rectus is sometimes used to reduce contracture, for a review see Escuder and Hunter 2019.(Escuder and Hunter 2019) Repetitive eye movement activities have not been studied for their potential ability to reduce contracture, yet this is one mechanism by which repetitive oculomotor exercise (neuro re-education) may be beneficial. These “ocular range of motion activities” (repetitive pursuits and saccades to the end of the range) were already commonly being utilized for this purpose by OTs in our facilities. The approach is supported by current understanding of extra-ocular muscle physiology. For example, in order to obtain typical high eye-movement saccadic velocities, the EOMs are tonically innervated at rest. With every attempted saccade, a coordinated excitatory “full-on” impulse to the agonist and reciprocal inhibitory “full-off” switching occurs (referred to as Sherrington’s Law, for a historical review see Ciuffreda and Stark, 1975.(Ciuffreda and Stark 1975) The duration of the reciprocal event is dependent upon the planned amplitude. Therefore, there may be a benefit to large (>20°) and repetitive saccades to repeatedly and maximally relax the antagonist while actively stretching with any residual agonist function. The next question asked was whether there should be an attempt to hold the eye at the eccentric position after each saccade (gaze holding). Once the eye reaches its intended target, tonic innervation to the antagonist returns, and so in the case of OCNP, there is often difficulty sustaining this eccentric gaze (personal observation). The eye drifts toward the neutral position until a stimulus generated saccade is initiated in an attempt to regain fixation. This process will cycle during the attempted eccentric gaze holding, which may provide some benefit for contracture prevention and neuro re-education. The suggested duration of gaze holding between repetitive saccades was set at 1-2 seconds. To summarize, the combination of antagonist inhibition and stretching via any spared agonist action should be maximized by repeated fast-movement full range saccades with a hold at the end. In our experience this approach is not uncommon among OTs and OD vision therapy specialists. In our protocols we refer to these as repetitive saccades (Appendix 2 and Figure 3.B.1).

**Discourage Disuse of the Paretic Extra-Ocular Muscles:** “Disuse” syndromes are well described in upper extremities affected by stroke.(Wolf, Lecraw et al. 1989) By encouraging use of the paretic extremity via constraint-induced therapy, cascading neuronal death (apoptosis) was prevented in animal models (Qu, Zhao et al. 2015) and benefits gained at the behavioral and physiological level in human patients (Kwakkel, Veerbeek et al. 2015). **This concept has never been studied in the oculomotor system** likely due to the potentially flawed clinical rationale that the eyes are continually moving, even when under monocular occlusion. The continual movement argument is not well supported when considering Herring’s law, which asserts that there is a single impulse signal (controller) for both eyes and, therefore, innervation to the eyes for conjugate gaze cannot be modulated independently.(Leigh and Zee 2015) If Herring’s law is correct, when the unaffected eye directs an accurate and appropriate conjugate eye movement, the affected muscle in the opposite eye receives less impulse due to lost nerve conduction from the injury. This would be the case whether the affected eye is occluded or in binocular viewing, and would represent a form of disuse, contributing to the atrophy documented with MR imaging in such cases.(Demer, Ortube et al. 2006) Compensatory head posturing may also contribute to disuse, under binocular or monocular viewing, by limiting the frequency and magnitude of eye-in-orbit movements towards the field of the affected muscle. Such disuse might be prompted to avoid diplopia, or because of a lack of efficiency of eye-relative-to-head movements during tasks requiring repeated gaze shifts in the paretic direction. We have also observed this compensatory posturing behavior under monocular viewing with the paretic eye, suggesting the behavior may not be entirely related to maintenance of single vision. We next wondered if there was rationale for the occlusion of the unaffected eye to encourage use of the paretic eye to promote recovery. When occluding the unaffected eye and requiring fixation with the paretic eye, a larger range of motion of the affected eye should be possible under Herring’s law, by brainstem controller upregulation of phase and step. According to Herring’s law, this would cause relative over-action in the unaffected eye, a well-known phenomenon most easily observed in internuclear ophthalmoplegia as an abducting nystagmus.(Leigh and Zee 2015) Therefore, if range of movement of the paretic eye is increased in magnitude by occluding the unaffected eye, this may reduce disuse and eventual contracture, while also promoting saccade and vestibular ocular adaptation, for a review of recommendations on translation of concepts from laboratory to clinical practice see Zee 1994 and Schubert and Zee 2010.(Zee 1994, Schubert and Zee 2010) Existing common practice by OTs on our neuro-rehabilitation units for patients whom require patching (monocular occlusion) to manage diplopia symptoms, was to alternate the eye that was patched (usually daily). This approach would be supported by the preceding rationale when palsy is subtotal. However, there were potential problems with alternate patching which were considered by the SIG. Firstly, the patient would have to re-adapt daily which would be uncomfortable and likely disorienting, and they may be less functional when using the paretic eye, or have a higher risk for fall. It is also possible (if not very likely) that even with patching of the unaffected eye to eliminate diplopia in the direction of the paretic motor field, the patient would adopt a head posture which limited the need to move the eye in the paretic direction (however, no prior evidence was available to guide the decision). As such, it was reasoned that focused sessions for the affected eye, with monocular repetitive oculomotor exercise with head stabilization, was a better approach than alternate patching. An exception was also considered, in the case of total palsy, where occluding the non-paretic eye would not increase the range of the affected eye (the nerve simply cannot provide any impulse to the muscle). Use of repetitive exercise in a complete palsy would not be expected to improve range of motion.

**Promote Recruitment of Nearby Musculature:** All of the extra-ocular muscles have secondary and tertiary actions dependent upon the position of the globe in the orbit.(Von Noorden 1983) For example, the superior recti elevate the eye when it is in a neutral primary gaze position, but provide increasing intorsion with increasing adduction. Therefore, the superior rectus might be recruited for the loss of intorsion caused by 4^th^ nerve palsy, by adopting a head turn toward the affected eye. Partial activation of EOMs is a different consideration. The degree to which this occurs in normal conditions versus what is possible after pathology is a knowledge gap area.

**Promote Adaptive Re-Balancing of Conjugate Eye Movement with Binocular Sensorimotor Therapies:** If Herring’s law is accurate, the oculomotor system has no mechanism within the conjugate eye movement network by which to balance the disturbed innervation to one eye caused by unilateral OCNP palsy. However, Herring’s law is part of a century old scientific controversy between Herring and well-known ophthalmologist, Von Helmholtz, who instead argued for the existence monocular controllers of conjugate eye movement, for historical review see Ebenholtz 1970.(Ebenholtz 1970) Earlier neurophysiological studies supported Herring’s law; however, more recent evidence has accumulated to support independent control is possible when needed (for a review see King 2011 (King 2011) and for more recent work Maiello et. al.(Maiello, Harrison et al. 2016) Independent uniocular control mechanisms (impulse generators and integrators), if available, would allow the vergence and conjugate gaze system to adapt to monocular nerve palsies even without full recovery of the nerve, so long as the injured nerve or nuclear complex is not completely damaged. Such an adaptive rebalancing process would rely on feedback from each eye (Binocular Sensorimotor Loop), and therefore would be inhibited by the use of a patch and potentially enhanced by binocular sensorimotor therapies.

**A1.1c.2 Techniques for Oculomotor Neuro Re-Education:** During the implementation of the new protocols, most OTs working on our rehabilitation units (authors included) were already incorporating repetitive eye movement re-training activities into their treatment sessions. This included visual tracking (pursuit) to e.g., pen target and saccades (eye jumps) between laterally displaced targets. While there were positive anecdotal reports, there was little evidence available to support the efficacy of the approach. Rather than removing these activities, the OD-OT SIG leadership sought to standardize the methodology and implement additional techniques based on current understanding of the oculomotor pathways and disease states, as illustrated in Figure 3. In general, eye movements may be 1) reflexive via the midbrain collicular attentional system (superior colliculus, (SC)); 2) reflexive via vestibular system input directly into the oculomotor saccade generators; 3) pre-conscious via visual-stimulus driven processes arising from parietal eye field (PEF) input to the superior colliculus; or 4) “generated by the will of the patient”(von Helmholtz 1962) (goal-directed, volitional, top-down) arising from activity in the cortical networks in the cingulate and frontal eye fields.(Leigh and Zee 2015) In performing repetitive volitional (willful) saccades or visual tracking, OTs had been utilizing primarily top-down (cortico-collicular) pathways for oculomotor re-education. Bottom up approaches might be more feasible for brain-injured patients as the reflexive-preconscious nature minimizes the need for the patient to understand, follow and self-initiate the eye movements. **Alternate Cover Activity:** In OCNP, when the fixating unaffected eye is covered, the affected eye will saccade in the paretic direction in an attempt to regain fixation(Figure 3B.2), a preconscious process utilizing the parieto-collicular pathway, PEF to SC (Figure 3D). The cover paddle can be alternated between the eyes to repeat this process, after which the cover is removed and a fusion eye movement may occur (Figure 3.D.4). Alternate cover is a well-known and studied technique for diagnosing and measuring strabismus, but when done repetitively, might also act as a therapeutic intervention for OCNP. Alternate cover has long been utilized as a binocular vision therapy technique to break suppression (see page 20 of the Pediatric Eye Disease Investigator Group Randomized Trial Comparing Patching with Active Vision Therapy Manual of Procedures.(Hopkins, Lyon et al. 2008) In our experience, it also stimulates fusional eye movements, possibly via motor priming (Stoykov and Madhavan 2015) and prediction, referred to as “forward models” (a cerebellar function),(Schubert and Zee 2010) and may result in dual adaptation such as that found to occur in adaptation of the eye-hand coordination loop to alternating prism exposure.(Welch, Bridgeman et al. 1993) The minimum requirement for the alternate cover activity was an ability to hold attention on the stimulus in approximately 20 second intervals, which was expected to be feasible for most patients in the acute care facilities (IRF1 & 2), and for some in the LTACH. Increasing the saliency of the stimulus (e.g. flashing penlight) and using auditory cueing could be helpful if sustaining attention was an issue. **Binocular Head Rotations Single-to-Double:** In this activity, a small fixation target such as a high contrast plus-sign was presented on paper or a blank tablet screen (Figure 3C) and the patient was asked if they could notice the double images occuring due to the strabismus. In the case of right 3^rd^ nerve palsy (Figure 3C.1), they were cued to turn the head to the left and tip back (Figure 2) while continuing to fixate the plus-sign, reporting when the double images fused. If the images never fused, and moving the target further away did not help (Figure 2), they were asked to get the double plus-signs as close together as possible. Then they were asked to slowly return the head to the neutral position or beyond until they noticed double images again (as illustrated in Figure 3.C.2) where the right eye failed to adduct, resulting in exotropia of that eye with the horizontal double vision of the plus sign illustrated in the middle panel. This was repeated up to 50 times, as tolerated. Right 6^th^ nerve palsy (Figure 3.C.3 and 4) would utilize a similar approach but in the opposite direction. While the primary purpose of binocular head rotations was to reinforce compensatory strategies, it did not exclude potential restorative neuro re-education effects on range of motion and binocular vision, and by promoting adaptive mechanisms in the vestibular ocular system (for a review, see Zee 1994 and Schubert and Zee 2010.(Zee 1994, Schubert and Zee 2010) Fairly recent studies in 4^th^ (Wong, Sharpe et al. 2002) and 6^th^ (Wong, Tweed et al. 2002) nerve palsy show the capacity for monocular adaptation of the VOR, again in conflict with the concept of a single controller as proposed by Herring, and supporting our approach described here, or alternatively, avoiding use of a patch solely on the paretic eye. Head rotation, unlike visual pursuit, recruits the vestibular ocular reflex (VOR) pathway (Figure 3.A.2), reflexively driving counter-rotational eye movements (Figure 3D, e.g., VIII to VI & III). Much like the alternate cover, this head rotation activity recruits bottom-up pathways. Even if the patient was unable to reliably report double vision, it was reasonable to expect potential benefit so long as fixation on the plus-sign could be sustained and the head rotated, either passively or actively. Rapid rotation (head-thrusts) would not require fixation, however, concerns about injury or pain from repeated treatment existed. The protocol also allowed for performance of the activity monocularly, for the potential to increase range of motion thereby discouraging disuse and contracture. It was reasoned that this activity could amplify the magnitude of the eye-in-head movement relative to the customary visual tracking without head movement. **Vergence eye movement activities** including pencil push-ups and Brock string, were incorporated into protocols. The 6^th^ nerve protocol concentrated on divergence and 3^rd^ and 4^th^ on convergence. Convergence eye movements are rapidly trainable with RCT-level evidence for convergence insufficiency in non-brain injured populations.(Scheiman, Kulp et al. 2020) The SIG felt it was reasonable to include both convergence and divergence activities based on existing clinical vision therapy practice patterns, personal experience of the authors, low cost, and low risk.

**A1.2 Homonymous Hemianopia Rehabilitation**

**A1.2a Background and Rationale:** Homonymous hemianopia is a loss of visual field in each eye on the side opposite the brain injury. Hemianopia may be incomplete involving only a portion(s) of the hemifield, or complete, and the depth of visual loss can range from a mild depression of sensitivity (relative defect i.e., scotoma) to absolute scotoma. Much like the physiological blindspot, when the field loss is absolute, the blind visual areas are invisible to the patient, which may create a problem with recognition and awareness. Another challenge may be the dynamic nature of hemianopia caused by the retinotopic representation, with the blind area(s) constantly moving, fixed to the largely unconscious eye movements. The best analogy is an afterimage or photo-stress scotoma from flash photography, except in hemianopia the scotoma is invisible. The dynamic nature may cause problems understanding the visual deficit for many patients. Patients often describe a variability of their vision, which may limit their ability to learn to compensate. The hemi-central field corresponding to the macula (within ~10° of fixation), which is important for most visual tasks, may be spared or split. When the peripheral field loss is incomplete or relative, it is likely easier for the patient to recognize that there is a visual problem (peripheral field is visible but of reduced brightness or clarity). It may also be easier to recognize where there is macular splitting, as attended objects would fall partially into the scotomatous area. In low vision rehabilitation evaluation, it is typical to classify any spared vision by functional ability such as 1) motion detection (hand-motion perception), 2) accurate localization motion or forms (projection), or 3) accurate form perception (e.g. able to count fingers). The visual loss can be identical in each eye (congruent) or somewhat less in one eye (incongruent). In incongruent cases, the better eye typically defines the need for rehabilitation, as patients live their lives under binocular conditions.

Prisms which sit in a portion of the lens (sector prisms) were offered/demonstrated to all patients who had field loss which seemed to be causing obstacle detection failures during ADL training with the OT, or when at least one quadrant had field loss at the level of hand motion without projection, or worse. The peripheral prisms design, marketed as the Peli lens, (Chadwick Optical, Harleysville, PA) was exclusively used based on strong evidence base to improve field of view up to 40° which in-turn improved mobility (see results from a double-blind multicenter RCT).(Bowers, Keeney et al. 2014) Other sector prism designs such as Gottlieb visual field awareness system (X-treme Optics, Lithia Springs, GA) and Hemilens (Chadwick Optical, Harleysville, PA) were also considered. However, there were conceptual and optical limitations to other sector prism designs which may render them less effective and in some cases, could actually reduce safety as described in detail previously.(Apfelbaum, Ross et al. 2013) Fitting of Peli lens essentially always adhered to the manufacturer fitting protocol, to straddle the border of the field defect in primary gaze with a press-on 40^∆^ sector prism above and below the line of sight over the eye on the side of the defect, in primary gaze.

| 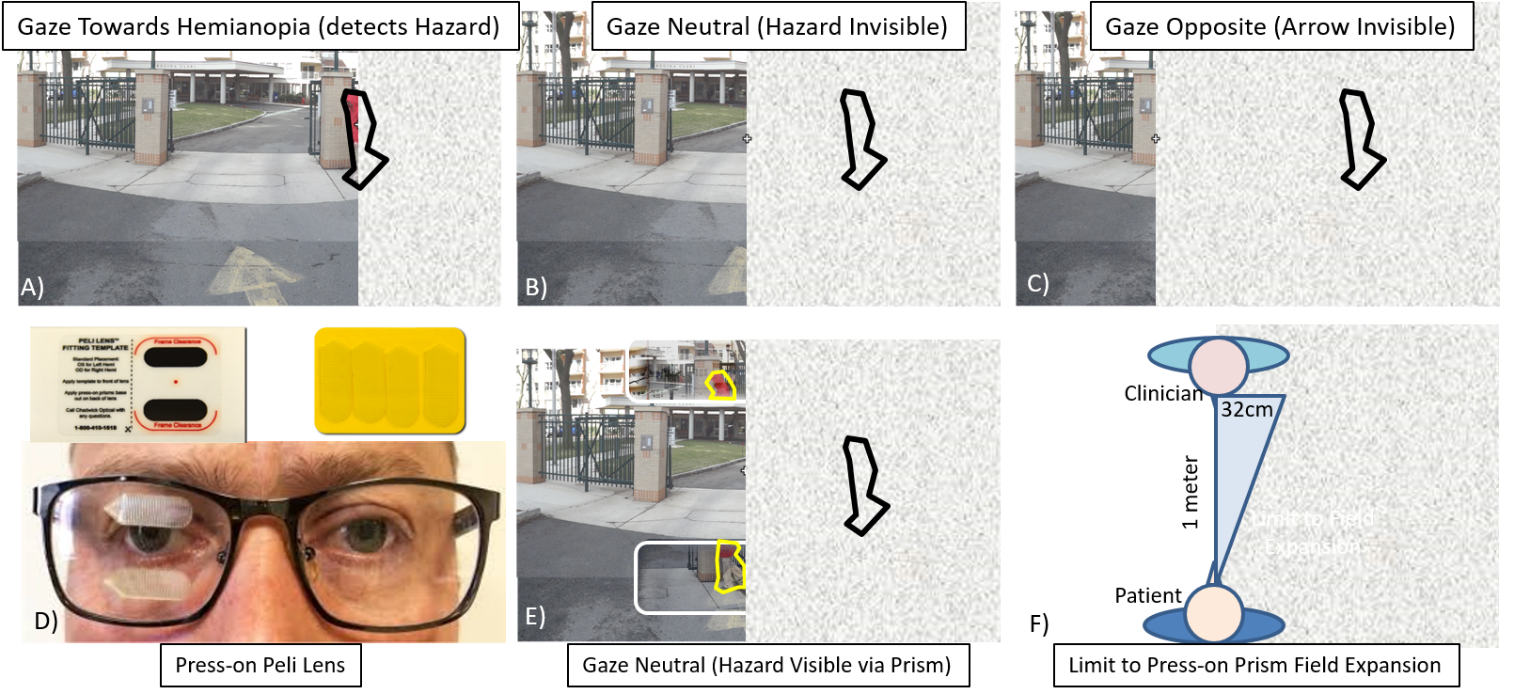 |
| --- |
| Figure A2: (A-C) Photo-simulation of a patient point-of-view in complete absolute right homonymous hemianopia, illustrating the retinotopic nature. Illustrations were adapted from those used in staff training seminars. The reader should imagine standing in-line with the center of the driveway just behind the yellow traffic arrow. The white cross corresponds to the point of fixation (fovea), the noise pattern represents the blind hemifield, and the black outline shows where the pedestrian hazard is located within the blind field. Notice the visual loss moves with the fixation point, and in (C) when fixating to the side opposite the hemianopia, the patient cannot see what is directly in front of them, such as the traffic arrow and the driveway, representing the greatest fall risk situation (should an obstacle be in the path of walking). (D) 40 prism diopter (18°) Peli lens, standard fitting for right hemanopia, available in the U.S. only via Chadwick Optical, Harleysville PA. (E) Simulation of patient binocular view with Peli lens when gazing straight ahead, compare to (B). The pedestrian hazard is visible via the prism, outlined in yellow for emphasis. Note that the simulation was for the 57 prism diopter oblique design, which gives more field of view expansion than the press-on version. F) OTs were provided education with this simulation illustrating the limits to the field of view expansion. When the OT-clinician was seated across from the patient at 1m, the prisms would only extend the field to approximately the outside the shoulder. |

Evaluation for hemianopia was offered in cases where there was suspected field loss. The hemianopia vision rehabilitation protocol was typically offered when the field loss was impacting ADLs per the OT, or presumed to by the OD and/or OT based on the degree of hemianopia, awareness of the visual loss, and demonstration of compensatory behaviors such as the ability to quickly locate peripherally placed objects with eye and head scans. Much like binocular and oculomotor rehabilitation, hemianopia rehabilitation interventions were categorized as primarily top-down (compensatory scanning) and bottom-up (Peli peripheral prisms). Prior to this study, our facilities were exclusively utilizing top-down approaches and verbal education with non-standardized definitions of hemianopia and varying levels of understanding of the disorder across care providers. Attempts at hemianopia restoration therapy via repeated stimulation of the border of the visual field and via perceptual learning (Dundon, Bertini et al. 2015) have been studied with substantial debate surrounding to their efficacy (see Schofield and Leff 2009 for objective discussion of the debate.(Schofield and Leff 2009) A commercially available product, NOVA vision Inc., Vision Restoration Therapy (Boca Raton, FL), was and continues to be available. However, it is designed for home administration over several months and due to high demands on visual attention and time (holding fixation for 15-25 minutes twice a day 6 days a week), was not likely to be feasible for our acute patient populations. More recently, studies of perceptual learning (Huxlin, Martin et al. 2009) and audio visual stimulation (Grasso, Làdavas et al. 2016) have been proposed and studied, but even at present these therapies are not commercially available.

**A1.2b Hemianopia Rehabilitation Protocol**

1. Fitting of Peli Lenses: Lenses were fitted according to the manufacturer’s instructions (Chadwick Optical, Harleysville, PA) when visual field loss was grossly complete and any residual vision in the affected field was not better than hand-motion without projection. Some exceptions were made, fitting patients with projection or partial finger counting, based on OT report of impaired detection of hemianopia side obstacles. The OD performed the initial prism education training as has been described in prior clinical trials, asking the patient to notice the examiner’s hand in the prism field of view, and notice the prism image shift. Patients were instructed to not look directly into the prisms, and instead shift their gaze to the hemianopic side to visually inspect any obstacle detected via the prisms. OTs were tasked with re-enforcing the Peli lens concepts and monitoring safety with the Peli lens and retained the option to remove the prisms if they perceived any safety issue or if poor tolerance was interfering with participation in therapies.
2. Compensatory Scanning Training: Due to the retinotopic nature of hemianopia, there is little debate concerning the compensatory value of frequent gaze shifts to the affected side. That said, controversy does exist between experts as to whether it is actually necessary to train patients to scan, as they may develop such skills on their own. The most common training technique we had seen employed was cueing to look to the affected side during ADL training. This type of training was already frequently being utilized on our rehabilitation units with reported benefits by most of the OT vision SIG participants. The cueing method trains scanning directly on the ADL task of interest, making this method more resistant to the well-known problem of generalization. Lack of generalization was a concern for more elaborate scanning training tools, such as scanning light boards (e.g. Dynavision (Bioness, Valencia, CA), Wayne Saccadic Fixator (Bernell VTP, Mishiwaka, IN), and visual search saccadic workbooks (Bernell VTP Mishiwaka, IN). However, these tools were already available in our facilities and regularly used by some OTs, and so were also mentioned as approved activities in our protocol.
3. Anchoring and Visual Search Strategies: Anchoring can be defined as *the use of a visual object at which a systematic scanning strategy would start and end*. Examples of appropriate anchors for mobility are a wall or baseboard on the side of the hemianopia, whereas for reading or visual search, the patient’s own thumb/finger or a solid line drawn down the column on the side of the visual field loss might be used. The goal was that the patient would be able to self-initiate selection of anchors in appropriate situations as a targeted reference point, for the most common ADLs, prior to discharge. Anchoring was part of the compensatory visual scanning training.
4. Patient Education and Awareness Training: An understanding of the vision loss would likely aid self- initiation of compensatory scanning and eccentric viewing and provide psychological benefit. This was accomplished by asking the patient to keep fixation on the clinician’s face and raise both arms, noting whether both arms could be seen or not. Ideally the patient would recognize, in the case of dense hemianopia, that they could not see the hand on the hemianopic side unless they glanced over. It was often demonstrated that the defect occurred in both eyes. It was unknown if provision of this education would improve understanding or lead to better outcomes. Efficacy determination was not the purpose of this project, but there was precedent. Awareness training in hemianopia is not novel to our approach, e.g. see de Haan et. al. 2015.(de Haan, Melis-Dankers et al. 2015)
5. Eccentric Viewing Training: Once the field loss was acknowledged, the impact of eye movement could be demonstrated, i.e. eccentric viewing training, which is frequently used in various types of visual field scotomas.(Li, Zhu et al. 2015) This involved demonstrating that the clinician seated directly in front of the patient was no longer visible when the patient was cued to adopt a fixation point about 40° away from the blind side. Conversely, an equal and opposite horizontal gaze offset, looking toward the hemianopic side but attending straight ahead, provided a more panoramic view of the room and full view of the examiner. Eccentric fixation concepts would then be reinforced during ADL training with the goal of demonstrating understanding prior to discharge.
6. Reading Rehabilitation: Hemianopia is most impactful on reading when the field loss is on the right and comes within 3° of the fovea, termed hemianopia alexia.(Schofield and Leff 2009) Right hemianopia is also closely associated with visual-cognitive reading impairments, such as pure alexia (impaired word recognition) for which patients were assigned to e Speech Language Pathology service for further rehabilitation and thus not managed within the vision rehabilitation inpatient service. Protocols for inpatient hemianopic dyslexia reading rehabilitation were entirely compensatory including a trial of 1) anchors in the column on the side of the defect, 2) use of finger to keep place, 3) use of a line guide, and 4) use of large print. Evidence for such compensatory approaches was poor and instead was based on clinical experience/consensus. Eye movement training with evidence based support (e.g. University College of London aphasia lab website (https://www.readright.ucl.ac.uk/) were reserved for outpatient administration,(Ong, Brown et al. 2012) as was training on assistive technology resources such as optical character recognition (i.e. screen readers).

**A1.3 Prism Adaptation Therapy Protocol for Left Hemispatial Neglect**

Prism Adaptation Therapy (PAT) is a visuo-motor rehabilitation technique for left neglect which utilizes ~10° (17.6 prism diopter) yoked prismatic goggles (e.g., Kessler Foundation, West Orange, NJ) which shift the entire visual field of both eyes rightward, temporarily dissociating the eye-hand coordination loop. The prismatic displacement causes the patient to mis-reach rightward; errors that were eliminated with practice in 50-60 trials (within a 2-minute session). The key component of the therapy was found to be the post-adaptation leftward aftereffect occurring after the prisms were removed. This induced leftward action bias was theorized to counteract the pathological rightward spatial errors in left neglect, with functional neuroimaging studies suggesting recruitment of left hemisphere cortical networks to account for the lost right hemisphere function.(Boukrina and Chen 2021) At the time of implementation, PAT was supported by two single center double-blind sham-controlled randomized clinical trials. (Serino, Barbiani et al. 2009, Mizuno, Tsuji et al. 2011) Another double blind RCT found short-term, but not long term, benefits.(Nys, de Haan et al. 2008) There was one negative result single-blind RCT, however an incorrect prism lens was used (10 prism diopters instead of 10 degrees (17.6 prism diopters), which we took to suggest that dose matters.(Turton, O'Leary et al. 2010) Some evidence was available suggesting generalization (transfer of effect) to activities of daily living unrelated to the therapy task, such as wheelchair driving, reading, and visual search. For a recent systematic review of effects on activities of daily living see Champod et. al. 2018.(Champod, Frank et al. 2018) There was also evidence to support a positive effect on the Functional Independence Measure,(Mizuno, Tsuji et al. 2011) which was the primary outcome measure for our IRF (and others in the U.S) at the time of implementation. Based on the existing evidence base for PAT, it’s excellent safety profile, simplicity (repetitive reaching), feasibility even for acute stage patients, and minimal equipment costs (about $300), PAT was added to our protocols for daily administration by OT. Other sensory stimulation techniques have been used to reduce left neglect (optokinetic, vestibular, caloric, tactile) and were also considered, but the effects were considered largely transient (for a review, see Priftis et. al, 2013).(Priftis, Passarini et al. 2013)

**Supplementary References**

Goodale MA, Milner AD, Jakobson LS, Carey DP. Kinematic analysis of limb movements in neuropsychological research: subtle deficits and recovery of function. Can J Psychol 1990;44:180-95.

Harvey M, Milner AD, Roberts RC. Spatial bias in visually-guided reaching and bisection following right cerebral stroke. Cortex 1994;30:343-50.

Buxbaum LJ, Ferraro MK, Veramonti T, et al. Hemispatial neglect: subtypes, neuroanatomy, and disability. Neurology 2004;62:749-56.

Farne A, Buxbaum LJ, Ferraro M, et al. Patterns of spontaneous recovery of neglect and associated disorders in acute right brain-damaged patients. J Neurol Neurosurg Psychiatry 2004;75:1401-10.

**Appendix A2: Anonymous OT Survey**

**Instructions**

**About this survey (as distributed)**

This survey should take about 5-10 minutes to complete. It includes 10 questions addressing your satisfaction with the inpatient visual neuro-rehabilitation protocols that have been implemented in the (Name) Rehabilitation Network since 2015. Please keep in mind that your answers will remain anonymous if you answer the survey using the following link: <https://www.surveymonkey.com/r/WRWTK27>.

Part A of survey: <https://www.surveymonkey.com/r/WRWTK27>

Part B of survey: <https://www.surveymonkey.com/r/ZQFDRBJ>

As a reminder, the protocols that we are referring to are:

1. Protocol for 3rd Nerve Palsy

2. Protocol for 4th Nerve Palsy

3. Protocol for 6th Nerve Palsy

4. Protocol for Binocular Vision Rehabilitation Therapy

5. Protocol for Cortical Visual Impairment

6. Protocol for Visuomotor Therapy

7. Protocol for Hemianopic Rehabilitation Therapy

8. Protocol for Oculomotor Rehabilitation Therapy

9. Protocol for Prism Adaptation Therapy

For more information on the protocols, please visit: <https://www.dropbox.com/sh/4bau337g0fswxj5/AADtteqAIwrqaw0RbBa1udgca?dl=0>

**Survey – Part A**

1. How long have you been working for the Spaulding Rehabilitation Network as an occupational therapist?
   - 1. **< 1 year**
     2. **1-3 years**
     3. **3-5 years**
     4. **5+ years**
2. In the past year, where have you assessed/treated patients with neurological visual impairments? Select all that apply.
3. **Outpatient facility**
4. **Inpatient rehabilitation facility**
5. **Long-term acute care hospital**
6. **I do not assess/treat patients with neurological visual impairments**
7. **Other (please specify): _______________**
8. In the past year, how often did you use these protocols when they are applicable to your patients? Please select all that apply.

| **Protocol** | **Not applicable to my patients** | **Never** | **Sometimes** | **Regularly** | **All applicable patients** | **Another professional uses them with my patients** |
| --- | --- | --- | --- | --- | --- | --- |
| 3^rd^ nerve palsy |  |  |  |  |  |  |
| 4^th^ nerve palsy |  |  |  |  |  |  |
| 6^th^ nerve palsy |  |  |  |  |  |  |
| Binocular |  |  |  |  |  |  |
| CVI |  |  |  |  |  |  |
| Visuomotor |  |  |  |  |  |  |
| Hemianopic |  |  |  |  |  |  |
| Oculomotor |  |  |  |  |  |  |
| Prism adaptation |  |  |  |  |  |  |

1. Overall, how feasible were the protocols to deliver? Please answer both questions below (4.1 & 4.2).

4.1: How feasible were they for the clinicians to understand and administer?

1. **Not at all feasible**
2. **Somewhat feasible**
3. **Feasible**
4. **Very feasible**
5. **Not applicable**

4.2: How feasible were they for patients to attend to and follow the directions?

1. **Not at all feasible**
2. **Somewhat feasible**
3. **Feasible**
4. **Very feasible**
5. **Not applicable**
6. What kind of training did you receive to learn how to use the visual neuro-rehabilitation protocols described above? Select all that apply.
   - 1. **Weekend course**
     2. **Video recordings of the weekend course**
     3. **In person with Dr. Houston during treatment sessions**
     4. **Self-study of the protocols and the procedures manual**
     5. **No training was ever provided**
7. Please read each of the treatments in the left column below. In the past year, how many of your patients do you think experienced improvements in functional tasks performed binocularly as a result of receiving these treatments?

|  | **Not applicable to my patients** | **None** | **A few patients** | **Many patients** | **All applicable patients** |
| --- | --- | --- | --- | --- | --- |
| Prisms for correction of double vision |  |  |  |  |  |
| Peli lens for hemianopia field expansion |  |  |  |  |  |
| Using the Peli lens for reducing visual field cut behaviors |  |  |  |  |  |
| Prism adaptation therapy |  |  |  |  |  |
| Postural adaptation therapy |  |  |  |  |  |

1. Of your patients who received the Peli lens for hemianopia field expansion, how many of them were able to wear/tolerate it? How many of these found it helpful for reducing visual field cut behaviors? Please answer both questions (7.1 & 7.2).

|  | **0-25% of patients** | **25-50% of patients** | **50-75% of patients** | **75-100% of patients** | **Not applicable** |
| --- | --- | --- | --- | --- | --- |
| 7.1: Tolerated the Peli lens |  |  |  |  |  |
| 7.2: % For which it reduced visual field cut behaviors |  |  |  |  |  |

1. In the past year, in what percentage of cases was prism adaptation therapy helpful for patients with right neglect? For patients with left neglect? Please answer both questions (8.1 &8.2).

|  | **0-25% of patients** | **25-50% of patients** | **50-75% of patients** | **75-100% of patients** | **Not applicable** |
| --- | --- | --- | --- | --- | --- |
| Helpful for patients with **right** neglect |  |  |  |  |  |
| Helpful for patients with **left** neglect |  |  |  |  |  |

1. In the past year, what percentage of your patients with 3^rd^, 4^th^, or 6^th^ nerve palsies were treated using the postural adaptation strategies? For how many of these was it helpful in improving their functional vision? Please answer both questions (9.1 & 9.2).

|  | **0-25% of patients** | **25-50% of patients** | **50-75% of patients** | **75-100% of patients** | **Not applicable** |
| --- | --- | --- | --- | --- | --- |
| 9.1: % Treated |  |  |  |  |  |
| 9.2: % For which treatment was helpful |  |  |  |  |  |

1. In the past year, what percentage of your patients do you think benefitted from these treatments/protocols overall?

|  | **0-25% of patients** | **25-50% of patients** | **50-75% of patients** | **75-100% of patients** | **Not applicable** |
| --- | --- | --- | --- | --- | --- |
| % Benefited |  |  |  |  |  |

1. If you have any additional comments about the protocols, please write them below:

**Part B**

1. Which of the following strategies do you use for the visual problems listed below? Check all that apply.

|  | Field expanding prism (Peli) | Other prism | Postural adaptation strategies | Scanning – non-computerized | Scanning - computerized | Field enlargement training | Vergence exercises | Training for saccades, pursuits, fixation | Environmental modification | ADL training | Assistive devices (mags, typoscopes, etc.) | Environmental modification | Explanation/ provide info | Not applicable |
| --- | --- | --- | --- | --- | --- | --- | --- | --- | --- | --- | --- | --- | --- | --- |
| Visual field problem (hemianopia) |  |  |  |  |  |  |  |  |  |  |  |  |  |  |
| Visual neglect |  |  |  |  |  |  |  |  |  |  |  |  |  |  |
| Visual processing problem (Cortical Visual Impairment) |  |  |  |  |  |  |  |  |  |  |  |  |  |  |
| Eye movement disorder/diplopia |  |  |  |  |  |  |  |  |  |  |  |  |  |  |
| Other visual problem:  _____________ |  |  |  |  |  |  |  |  |  |  |  |  |  |  |

**Appendix A3: Protocols**

| **3^rd^ Nerve Protocol** |
| --- |

**Patient and OT Staff Education**

Explanation of diagnosis, prognosis (6–12-month recovery window), and postural modifications. Lack of evidence for postural strategies and oculomotor re-education exercises discussed. Option to decline treatment (choice) provided to patient and OT.

**Prism Fitting**

4^∆^ will be used Base IN and Up over the affected eye. Deviations from protocol at OD discretion, including use of 8^∆^, placing on unaffected eye. Deviations smaller than 4^∆^ will not receive prism. Deviations larger than 8^∆^ or where symptoms are not adequately managed will receive recommendation for patching with some combination of prism use during supervised OT sessions. OTs will hold authority for final discharge recommendation (prism/patch/combination/no treatment) based on observed response with safety and function as the primary determinants.

**3^rd^ Nerve Oculomotor Neuromuscular Re-education**

Frequency: A minimum of 3 sessions per week until goals are met, or patient is discharged.

**Head-Rotations Single to Double**

1. Red-green glasses may be used during the task to provide better feedback to the patient (but are not required)
2. Place a fixation target at distance (eg. small plus or “x”)
3. Cue patient to notice double images
4. Alternate cover to break suppression if necessary (de-clutter or provide larger or brighter target- see manual for detailed anti-suppression techniques)
5. Slowly turn the head VERY SLOWLY to move the images closer together (find head position where it is single).
6. Slowly return the head to neutral, verbalizing when the target blurs or doubles (repeat for several minutes).
7. Repeat, going double to single
8. Repeat at far or near

***Try to incorporate this strategy during ADL’s when the patient reports or is symptomatic of double vision.**

Tips: It usually takes 60-100 trials to achieve motor adaptation. If there is no perceived double vision, do the alternate-cover cover-uncover activity (see manual under anti-suppression activities). Also check that one eye is not closed or otherwise occluded.

**Alternate Cover, Cover-Uncover:**

1. Stabilize head.
2. Give a fixation target (e.g., small plus or “x” at distance or near).
3. Cover one eye then the other. Watch for the eye to move to pick up fixation.
4. Alternate 15 times or more and then uncover both eyes (teach patient to use the cover).
5. Cue the patient to “look hard” or “focus” or “cross the eyes” to bring the images together. Provide sufficient time to try. Cue not to close an eye.
6. Repeat 3 times

**Repetitive Visual Pursuit**

1. Cover the unaffected eye
2. Provide a small letter or plus target
3. Cue patient not to move their head or use an aid to stabilize
4. Move target as slowly as possible toward the affected direction(s)
5. Ask patient to report when they cannot follow it any longer or look for failure of eye tracking
6. Go back slowly cueing patient to try to recover
7. Repeat 50 times or until fatigue

**Gaze Holding**

1. Cover the affected eye
2. Cue the patient to look as far in the affected direction(s) as possible
3. Cue to hold for 2 minutes or until fatigue
4. Can provide a small letter, plus target, or series of letters to aid in sustaining gaze (optional)

**Brock String or pencil push-ups**

1. Fast Vergence (bead jumps): Repeated shift gaze
2. Slow Vergence (bug on string): Imagine bug walking down the string
3. Different positions of gaze: Primary gaze, down gaze, left gaze, right gaze

# PROTOCOL FOR 4TH NERVE REHABILITATION

**Patient and OT Staff Education**

Explanation of diagnosis, prognosis (6–12-month recovery window), and postural modifications. Lack of evidence for postural strategies and oculomotor re-education exercises discussed. Option to decline treatment (choice) provided to patient and OT.

**Prism Fitting**

4^∆^ will be used base down over the affected eye. Deviations from protocol at OD discretion, including use of 8^∆^, placing base up on unaffected eye, tilting obliquely in or out for horizontal components. Deviations smaller than 4^∆^ will not receive prism. Deviations larger than 8^∆^ or where symptoms are not adequately managed will receive recommendation for patching with some combination of prism use during supervised OT sessions. OTs will hold authority for final discharge recommendation (prism/patch/combination/no treatment) based on observed response with safety and function as the primary determinants.

**4^th^ nerve oculomotor neuro re-education protocol (see manual for detailed instructions)**

Frequency: A minimum of 3 sessions per week until goals are met, or patient is discharged.

1. Instruct patient to look at a small (~20/30) target such as a “+”.
2. Very slowly tilt the head to the right shoulder and back to neutral, verbalizing when the target blurs, doubles, or crisscrosses.
3. Stop and slowly tip the head back towards the shoulder trying to bring the images together.
4. Once the images fuse, slowly tilt the head back towards neutral.
5. Repeat this for 5 minutes or until fatigue.
6. Repeat for double to single
7. Repeat for turn head side to side

***Try to incorporate this strategy during ADL’s when the patient reports or is symptomatic of double vision.**

Tips: It usually takes 60-100 trials to achieve motor adaptation. If there is no perceived double vision, do the alternate-cover cover-uncover activity (see manual under anti-suppression activities). Also check that one eye is not closed or otherwise occluded.

# PROTOCOL FOR 6TH NERVE REHABILITATION

**Patient and OT Staff Education**

Explanation of diagnosis, prognosis (6–12-month recovery window), and postural modifications. Lack of evidence for postural strategies and oculomotor re-education exercises discussed. Option to decline treatment (choice) provided to patient and OT.

**Prism Fitting**

4^∆^ will be used Base Out over the non-dominant eye. Deviations from protocol at OD discretion, including use of 8^∆^ and an oblique tilt to the prism for vertical component common in 6^th^ nerve palsy. Deviations larger than 8^∆^ or where symptoms are not adequately managed will receive recommendation for patching with some combination of prism use during supervised OT sessions. OTs will hold authority for final discharge recommendation (prism/patch/combination/no treatment) based on observed response with safety and function as the primary determinants.

**Oculomotor Neuro Re-education**

Frequency: A minimum of 3 sessions per week

Head-Rotations Single to Double

1. Place a fixation target at near (e.g., small “+” or “x”)
2. Cue patient to notice double images
3. Alternate cover to break suppression if necessary
4. Turn the head VERY SLOWLY to move the images closer together (find head position where it is single).
5. Slowly return the head to neutral, verbalizing when the target blurs or doubles (repeat for several minutes).
6. Repeat, going double to single
7. Repeat at far

***Try to incorporate this strategy during ADL’s when the patient reports or is symptomatic of double vision.**

Alternate Cover, Cover-Uncover:

1. Stabilize head and give a fixation target (e.g., small plus or “x” at near).
2. Cover one eye then the other. Watch for the eye to move to pick up fixation.
3. Alternate 15 times or more and then uncover both eyes
4. Cue the patient to “relax” or “look or think big and far” to bring the images together. Provide sufficient time to try. Cue not to close an eye.
5. Its ok if they can’t fuse the images. It’s important to practice trying.
6. Repeat 3 times

Repetitive Visual Pursuit

1. Cover the unaffected eye
2. Provide a small letter or plus target
3. Cue patient not to move their head or use an aid to stabilize
4. Move target as slowly as possible toward the affected direction
5. Ask patient to report when they cannot follow it any longer or look for failure of eye tracking
6. Go back slowly cueing patient to try to recover
7. Repeat 50 times or until fatigue

Thumb Saccades

1. Patch the non-paretic eye
2. Stabilize head
3. Hold thumbs shoulder width apart and look right to left 50 times or until fatigue

Brock String Divergence

1. Move the beads out of the way
2. Notice where the strings cross
3. Keep looking for at least 5 minutes continuously
4. Notice any changes in the position where the strings cross
5. Goal is to learn to activate divergence eye movements to get the area of crossing to shift further back (divergence).
6. Repeat in 10 degrees left right and down gaze

# PROTOCOL FOR BINOCULAR VISION REHABILITATION

1. **Patient and OT Staff Education**
   - - 1. Explanation of diagnosis, prognosis (6–12-month recovery window), and environmental modifications. Option to decline treatment (choice) provided to patient and OT.
2. **Prism Fitting**

4^∆^ will be used at OD discretion. Deviations from protocol at OD discretion, including use of 8^∆^. Deviations larger than 8^∆^ or where symptoms are not adequately managed will receive recommendation for patching with some combination of prism use during supervised OT sessions. OTs will hold authority for final recommendation (prism/patch/combination/no treatment) based on observe response with safety and function as the primary determinants.

1. **Vergence Neuro-Muscular Re-education**
   - 1. Activities to promote fusion (seeing 1 image with both eyes open)
2. Alternate Cover, Cover-Uncover: Give a fixation target (e.g., small plus or “x” at distance or near). Cover one eye then the other. Alternate several times and then uncover both eyes. Patient may see double when cover is taken away. Cue them to try to fuse the images by either focusing or relaxing. Provide sufficient time to try. Cue not to close an eye.
3. Pencil Push-up: Slowly bring a target towards the nose while keeping eyes aligned (look hard, focus!)
4. Prism Bar Vergences: Ramp up vergence demand at about 1 prism diopter per second or so, “keeping it single!”
5. Brock string
   1. jumps (fast vergence): Look at the red bead, look at the green bead, look at the yellow bead, repeat….
   2. Brock string bug on a string (slow vergence): “Slowly walk the “x” (crossed strings) towards the nose and away.
6. Prism Jump vergences (flippers or loose lens ok): Put base out on, fuse, take it off. Repeat….
7. Near-far saccades: Look to close target (it should be double for a second). Look hard, make it single. Now quickly to distance, same thing.
   - 1. Transfer to ADL’s
        1. Using skills learned to promote fusion, attempt to transfer these skills during ADL training.
        2. Safety Assessment: After 1-2 weeks with fusion exercises and prism (if needed), do trials comparing safety under monocular (patching) vs. binocular conditions (using head posturing and prism as necessary).

# PROTOCOL FOR OCULOMOTOR REHABILITATION

Frequency: A minimum of 3 sessions per week until goals are met, or patient is discharged.

Indication: Vestibular-Oculo Reflex dysfunction, Saccadic Eye Movement Dysfunction, Pursuit Eye Movement Dysfunction

1. **Repetitive Voluntary Saccades**: Therapist or assistant stabilizes head patient’s head. Patient hold thumbs up at width of ears (substitute other targets if hemiplegia). Right, left, right, left: continue for ~60 trials, minimum of 3 times a week for 2 weeks.
2. **Gaze Stabilization:** (visual pursuit and VOR training)
   1. Head Rotations: Fixation “X” on patient’s bulletin board (or other similar target). Patient slowly rotates head left-right-up-down-tilt left-tilt right. Begin seated, move to standing when possible. Continue for 5-10 minutes, a minimum of 3 times a week for 2 weeks. Perform 1 cycle during ADL training in attempt to transfer effect.
   2. Eye Rotations: Head stabilized, patient holds fixation stick (pen, popsicle stick, etc.) and moves slowly (~3 deg/sec) left-right-up-down while trying to maintain fixation. Begin seated, move to standing when possible. Continue for 5-10 minutes, a minimum of 3 times a week for 2 weeks. Perform 1 cycle during ADL training in attempt to transfer effect.

# PROTOCOL FOR HEMIANOPIA REHABILITATION

1. **Patient Education and Awareness Training:**
   1. Patients with field defects most often are in denial that they fail to detect things, report they miss things only because they have been told so, rarely fully understand that they are blind in one half of each eye, usually think the eye on the side of the defect is blind, and don’t understand that the blindspot moves when they move their eye. As a result, they will complain that sometimes they can see, and sometimes they can’t.
   2. Discuss the cause of vision loss
   3. Personal Space Demo: Patient looks at examiner and holds up both hands (like being sworn into court).
   4. Examiner shows them how they can’t see their hand on the blind side unless they look over.
   5. Have them explore the edge of their blindspot with their own hand. EYES MUST REMAIN STATIONARY.
   6. Move from non-seeing to seeing
   7. Repeat moving from seeing to non-seeing
   8. Practice all along the edge of the defect for ~2min.
2. **Eccentric Viewing Training:**
   1. Once they recognize the blind field, demonstrate how it worsens when looking away from it, and improves when looking towards it. Continually ask the patient where the defect is on every visit multiple times.
   2. With the hands still up in the blindspot, have patient look to the wall to the side of the defect.
   3. Keep eyes still, pointed at the wall
   4. Instruct them to pay attention to the rest of the room being careful not to move the eyes.
   5. Ask if they can see most of the room and their hand that was previously in the blindspot in their peripheral vision.
   6. Instruct them to look to the opposite wall.
   7. Ask them to notice how the blindspot was drawn across the room like a shade.
   8. Ask them to notice how they cannot see you directly in front of them. Watch their eyes- they often scan and report they can see everything.
3. **Anchoring and visual search strategies**
   1. General Observation: “How much can you see in a room”.
   2. Right hemianopes should try stay to the same side of the room or hallway as the field cut. An immediate and significant improvement in their mobility will be seen. For left hemianopes this is not advised since it forces them to walk against traffic increasing risk of collision.
   3. Give an anchor 3-4 feet from them to continually scan back to (eg. Baseboard).
   4. When crossing threshold, patient should point eyes at door frame on the side of the field cut.
   5. Eventually force the patient to choose their own anchor.
   6. Saccadic Fixator/Dynavision: Place a piece of black tape on the edge of the device on the side of the field cut to act as a visual anchor. Practice returning to the anchor after each light is depressed.
4. **Reading Rehab**
   1. Black or red line page anchor on side of the field cut.
   2. Have patient use finger to guide eyes.
   3. Try line guides: Ruler or folded paper
   4. Trial large print

*Goal is for patient to employ any effective strategies without cueing prior to discharge

1. **Peli Lens Fitting**
   1. 40^∆^ press on horizontal prisms (Chadwick Optical, Harleysville, PA)
      1. For complete to near complete field loss (hemianopia affecting upper and lower quadrants to level of hand-motion without projection or worse, and OT report of detection failures during ambulation).
      2. Prism segments to straddle vertical meridian of visual axis in primary gaze (per manufacturer instructions, use of fitting template).
      3. At OD discretion, to break protocol, provide only an upper or low prism (e.g., frame too small, sparing of one quadrant).
      4. Peli lens (p-prism) demonstration training to be conducted by OD and reinforced by OT (see supplementary instructional sheet)
         1. Sections which patient cannot/should not participate can be omitted (i.e., walking up and down stairs)

**P-Prism Demonstration Training**

Peripheral prism glasses (p-prisms, aka Peli Lens or EP prisms) provide visual field expansion, easily documented on standard perimetry in patients with homonymous hemianopia (HH) (Peli 1998, Peli 2000). This approach employs monocular 40-57 prism diopter (20-25 degrees) sector prisms that are limited to the peripheral (superior and inferior) lens (Figure 1. c & d) but span both sides of the pupil, and thus expand the visual field at all lateral positions of gaze. Prisms of 40Δ were used with no difficulties in our first multicenter clinical trial (Bowers, Keeney, Peli 1998). The field expansion provided was found to be effective in helping patients with HH avoid obstacles while walking. Out of 43 patients with HH without SN, about 50% were still wearing the prism glasses after 12 months, reporting improved mobility. While p-prisms expand the accessible visual field immediately on first application, the perceived direction of objects detected through the prism is incorrect; objects on the blind side appear to be on the seeing side. For this reason, patients receiving p-prisms must be trained to use them.

Summary of how the prisms should be used

- In many ways training and adaptation resembles that required of first-time bifocal wearers, where head movements are needed to eliminate the blurry appearance of objects seen through the wrong part of the lens.
- Patients should be taught to look through the central prism-free area of the lens at all times.
- The patient should never look into the prisms otherwise they will experience central diplopia (double vision), which is disorienting and uncomfortable.
- The prisms act in peripheral vision. When an object of interest is detected via the prism, the patient should then turn his/her head and eyes to directly view the object with central vision through the central, prism-free, part of the lens.
- Objects detected via the prisms will appear to be shifted towards the seeing side. The patient will have to turn his/her head and eyes towards the blind side in order to view the object with central vision.
- These head and eyes movements require deliberate attention at first and should become almost automatic following training and practice.

Adjusting the Prism Glasses

- Adjust the **vertical** position of the prisms by first pushing the glasses up or down on the nose so the prism-free area is centered on the pupil. Adjust the nose pads as a last resort.

Demonstrate the visual field expansion effect of the prisms

- This procedure is similar to confrontation visual field testing, but is done under binocular viewing conditions (i.e., with **both** of the patient’s eyes open).
- Face the patient at eye level from a distance of 20 – 30 inches.
- Ask the patient to look directly at your nose through the center of the lens and NOT THROUGH THE PRISMS.
- With the patient still looking steadily at your nose, now move your hand in from the blind side into an area covered by the (upper) prism. Bring your hand in along a line about 2 inches above the pupil center (the horizontal midline). Ask the patient to report when they can detect your wiggling fingers. Your fingers should first be detected when still in the blind field, about 8 inches (25°) from the vertical midline. Repeat this for the lower field. The patient should be able to detect the movement in their peripheral vision, upper and lower, via the prisms while looking through the center of the lens, NOT THROUGH THE PRISMS.
- Demonstrate to the patient the difference between where the fingers were first detected without, and then with the aid of the prisms, by asking the patient to look directly at the fingers in each case through the prism-free portion of the lens.

Practice turning head and eyes to directly view object on blind side

- With the patient looking steadily at your nose, move your fingers again in the blind field in an area covered by the prisms.
- When the patient detects your moving fingers in the prism tell them to then look directly at your fingers by turning their head and/or eyes towards the blind side to view your fingers through the (prism free) center of the lens.
- Repeat this 60-100 times with your fingers in different positions for the upper and lower prisms.

Demonstrate undesirable central diplopia when patient looks directly through prism

- Ask the patient to look directly through either the top or bottom prism.
- By necessity, he/she will also be looking through the non-prism portion of the other lens. This will cause CENTRAL diplopia. Tell the patient that this is to be avoided.

Reach and touch – real and apparent positions of objects detected via prism

- This exercise will increase the patient’s understanding of where objects detected via the prisms really are in relation to where they appear to be.
- With the patient looking at your nose, move your fingers in the blind field in an area covered by the prisms.
- Ask the patient to grab at your finger as soon as he/she detects it through the prism while still looking straight at your nose.
- Chances are that the patient will miss your finger on the first try. He/she will tend to grab at the apparent position of the finger, which is closer to the seeing field than the real position. Explain this to the patient – explain that objects detected in the prism will seem to be closer to the seeing side than they really are.
- Have the patient repeat the exercise 60-100 times for each prism zone.
- If the patient is unable to perform this task within five minutes of practice (during the dispensing of the prism glasses) they will not qualify for the study.

Training walk

- Take the patient for a walk along a hallway and through uncluttered rooms. **The patient should be escorted at all times.**
- Progress to more cluttered rooms such as a reception area with tables and chairs. Finally, take the patient up and down a set of stairs.
- The training walk should ensure that the patient understands how the prisms give an indication of obstacles on the blind side.
- When a patient detects an object on the blind side (e.g. door frame, chairs, tables) from one of the prisms, he/she should be instructed to look directly at the object through the prism-free portion of the lens.
- Ask the patient to walk up and down some stairs. Walking upstairs should present no problem. When walking downstairs the patient should be taught how to avoid looking through the lower prism. Either the head will have to be lowered to see the stairs through the central prism-free area, or the patient will have to look underneath the lower prism. Similar problems are encountered by bifocal wearers and will require an adaptation period. Advise the patient to use handrails whenever possible.

**Prism Adaptation Therapy Protocol**

**Indications:** Left Hemispatial Neglect with Right Frontal, Temporal, Parietal, and/or Occipital Lobe Injury

**Equipment Needed:** Base Left 17.6PD (10°) Prism Adaptation Glasses. Ok to substitute 20∆ (11.4°) prism adaptation goggles.

**Step 1 Task Set-up and Practice:**

- Place two targets (eg. small pieces of tape) 1-2 inches left and right of midline
- Have the patient place their right hand at their midline (navel or sternum) and left hand on their lap
- Instruct the patient to “look at the target and reach quickly to touch it”
- Instruct the patient to return their arm to the starting position at their sternum after each trial
- Practice until they seem to understand the task

**Step 2 Adaptation:**

- Have patient close eyes and not open until instructed to
- Place the prism glasses on the patient, bases (thick side) to the left.
- Have the patient open the eyes and immediately begin the reaching task. The starting position needs to be with the arm OUT OF VIEW AT THE MIDLINE. It is very important that the arms are not visible until they are reaching toward the target. Feeling around or trying to correct for reaching errors after the arms are visible needs to be avoided in order to get the full effect of the therapy.
- Using the right arm, instruct the patient to quickly reach and touch the target 100 times. The therapist should instruct the patient at random to the left or right targets. The clinician should watch for the expected reaching errors to the right of the target, with gradual correction over 10-20 trials.
- 100 targets should be achieved before moving on to the next step.
- If adaptation is not achieved even with 100 targets, continue until the patient appears adapted or becomes fatigued.
- Record number of targets and successful adaptation.
- The therapy should not be stopped short of 100 trials because the patient “appears adapted”. Continued reaching maximizes the desired aftereffect

**Step 3 Post Test and measurement of realignment:**

- Have patient close eyes and not open until instructed to.
- Remove the prism glasses – keep eyes closed
- Have the patient open the eyes and immediately begin the reaching task again. The starting position needs to be with the arm OUT OF VIEW AT THE MIDLINE.
- The patient should miss left of the target. Note the magnitude of the initial miss in degrees.
- Note the number of trials to again reach the target accurately.

Other Considerations: The ideal therapeutic regimen is **2 sessions daily for 2 weeks**. Some patients will be unable to follow the protocol due to cognitive limitations or motor limitations. Patients who are unable to reach are unlikely to benefit from this therapy.

**Protocol for rehabilitation of visuomotor coordination problems**

**Indications:** Optic Ataxia, Cerebellar Ataxia, Oculomotor Apraxia, hemispatial neglect

**Common Symptoms:** Consistent Misreaching

**Equipment Needed:**

- Two or more targets

**Frequency: Minimum of 3 times/week x 2 weeks**

1. Repetitive t-VMT training (t-VMT= terminal^[[1]](#footnote-1)^ visuomotor task)
   1. 2 stickers on a table 1-2 in. right and left of midline, or with Dynavision or other similar visuo-motor board
      1. Occlude the patient’s view of their hand and body in the starting position at the sternum
      2. Have the patient reach quickly with the right hand to targets.
      3. Bring the hand back and repeat for ~60 trials, randomly alternating between targets.
      4. Repeat for the other hand if possible
      5. Repeat using common objects or incorporate into ADL training
2. Closed loop visuomotor training (online corrections)
   - 1. Using a pipe cleaner shaped into a loop (adjusted diameter based on the ability of the patient).
     2. Instruct the patient to make a pointer finger and keep their finger in the center of it as you move it.
     3. Continue for 2 minutes
     4. Repeat for the other hand if possible
3. Gaze-reach dissociation training
   - 1. Can be terminal reaching condition or open loop
     2. Patient encouraged to reach for objects without foveating them

Protocol for Rehabilitation of Cortical Visual Impairment

**Indications:** Reduced visual acuity or problems with object and text identification attributable to cortical injury.

Examples of Conditions: Visual agnosias, Bilateral hemianopia, Simultagnosia

**Equipment Needed:**

| 1. Patient’s glasses or loaner glasses | 2. Black Marker Pen |
| --- | --- |
| 3. White or off-white paper | 4. Black construction paper |
| 5. Goose neck lamp | 6. Foam or block letters |
| 7. Picture flash cards | 8. Common household items |
| 9. Report with minimum print size requirements |  |

Activities and modifications will depend on type and level of impairment. Listed here are the approved activities:

1. Letter identification with block letters

2. Trace and say letters and symbols

3. Word identification with block letters

4. Trace and say words

5. Identify objects (hold and touch)

6. Identify objects, flash cards (error free learning)

7. Identify objects, flash cards (see and say)

Compensatory techniques: Train the patient to adapt their own environment to maximize performance

1. Contrast enhancement: E.g., black placemat, direct lighting with goose neck lamp, labels with large black on white or white on black print. Use symbols on the labels instead of words or letters, if this is easier.
2. Adaptive technology: E.g., reading scanners, CCTV, reader pen, iPad/pod/phone aps
3. Line and finger guides: E.g., fold or cut a window in construction paper to isolate objects of interest.
4. Tactile/auditory mods: tactile stickers (on phone, label 911 with tactile stickers), system for money identification (e.g., 5’s folded in half, right corner folded for 10’s)
5. Anchoring and scanning to the worse side.

**Appendix A3: OT vision screen protocol**

**Occupational Therapy Department**

**Policy & Procedure**

# CLINICAL VISION EVALUATION PROTOCOL

**Abstract:** This protocol describes how to perform the OT Clinical Vision Evaluation.

**Level of Personnel:** All Occupational Therapists

**Description:** The purpose of the Clinical Vision Evaluation is to identify following:

1. Reduced reading ability
2. Oculomotor dysfunction
3. Strabismus (Eye Misalignment)
4. Visual field cuts
5. Hemispatial neglect
6. Any functional limitations possibly related to vision deficits
7. Other eye/vision problems

**Common Symptoms of visual disturbance:**

1. Closing an eye
2. Abnormal head posture (tilting, turning)
3. Bringing items close to see
4. Blur or double vision
5. Misreaching or poor depth perception during ADL’s
6. Veering or bumping when ambulating

Refer to this document for detailed vision testing instructions.

If the patient has a visual disturbance contributing to functional impairment, they should be scheduled for a vision evaluation with the inpatient Brain-Injury Vision Rehabilitation Specialist (BIVRS). The BIVRS will be a Doctor of Optometry or Ophthalmology with specialty training in brain injury vision rehabilitation and therapy. If indicated, the BIVRS will help you develop a plan of care and may ask you to complete a short survey to monitor vision rehab outcomes.

1. When visual deficits are identified, this should be reported to the attending doctor to obtain order vision clinic with BIVRS (OT order - vision consult).
2. Upon evaluation in clinic, the BIVRS will decide:
   1. If focused vision rehabilitation is indicated
   2. If further evaluation is indicated with an outside Ophthalmology Specialty Service.
3. The BIVRS will select appropriately from the approved vision rehab treatment protocol(s) and demonstrate proper techniques to the OT.
4. The OT’s responsibilities during vision treatment:
   1. Provide the prescribed treatment (see treatment protocols)
   2. Verbally convey diagnosis and treatment plan to the attending and other team members.
   3. Provide updates to the team as visual function changes
   4. Communicate any changes or protocol deviations with the BIVRS
5. Prior to discharge:
   1. Review continuum of care plan and reinforce to the patient, caregiver, and team
   2. Review recommendations for continued use of any prisms or vision therapy after discharge

**Criteria for Evaluation**

This evaluation can be reliably administered to patients who can:

1. Attend to test for at least 10-15 minutes.
2. Comprehend directions and follow 1-2 step commands.

**Procedure for Evaluation/ Documentation**

1. Occupational Therapy staff should be trained on the Clinical Vision Evaluation prior to administering.
2. Clinical Vision Evaluation is completed and noted in daily documentation under the Cognitive section.

Indicate diagnosis-

Test Screening items:

1. Visual Acuity (Near or far)

1. Distance- Have patient stand 10 ft. from large Snellen Chart. Cover one eye at a time, then test both eyes together.
2. Place “pocket-sized” Snellen Chart 16” from midline of patient’s face. Use the letters starting with the largest and work down consecutively. Cover one eye at a time, then test both eyes together.
3. *Refer to BIVRS with best corrected visual acuity worse than 20/40 in either eye or asymmetry between the eyes greater than 1 line.

2. Fusion/convergence: Observe the eyes in straight ahead gaze at distance and near for any misalignments. Refer to BIVRS vision clinic with any suspected misalignments.

3. Ocular Motility

1. Fix and Follow (pursuits): A pen is moved ~3deg/sec horizontally while observing the eyes. Refer with any suspected dysfunction.
2. Voluntary Saccades (fast eye movements): Repeated fast horizontal scans between 2 targets (examiner’s pen and finger) set at the width of the patient’s ears. Refer with any suspected dysfunction.
3. Range of Motion (ROM): Instructions to look left, right, up, and down are given while examining for any disconjugate movements (eye not moving together). Refer with any abnormalities.

4. Visual Fields

5. Reading Ability: Assess the patient’s ability to read standard and large print with their best correction.

**Clinic Evaluation Appointment (with the BIVRS)**

Please come with your patient to the vision clinic area at the specified time with the following items:

1. Glasses or other visual aids

If the patient is unable to leave the room, this should be indicated when making the appt. for clinic.

Vision Rehabilitation

The BIVRS will provide a treatment protocol to the OT including:

1. Vision therapy activities
2. Training in compensatory strategies

1. Terminal Reaching (def). Hand is not visible until the end (terminus) of the reach. [↑](#footnote-ref-1)
